# Overcoming Resistance to Temozolomide in Glioblastoma: A Scoping Review of Preclinical and Clinical Data

**DOI:** 10.3390/life14060673

**Published:** 2024-05-24

**Authors:** Dimitra Smerdi, Myrto Moutafi, Ioannis Kotsantis, Lampis C. Stavrinou, Amanda Psyrri

**Affiliations:** 1 Department of Medical Oncology, Second Department of Internal Medicine, “Attikon” University General Hospital, Athens Medical School, National and Kapodistrian University of Athens, 11528 Athens, Greece; 2 Department of Neurosurgery and Neurotraumatology, “Attikon” University General Hospital, Athens Medical School, National and Kapodistrian University of Athens, 12462 Athens, Greece

**Keywords:** glioblastomas, temozolomide resistance, resistance mechanisms, chemoresistance, therapeutic agents

## Abstract

Glioblastoma (GB) is the most common and most aggressive primary brain tumor in adults, with an overall survival almost 14.6 months. Optimal resection followed by combined temozolomide chemotherapy and radiotherapy, also known as Stupp protocol, remains the standard of treatment; nevertheless, resistance to temozolomide, which can be obtained throughout many molecular pathways, is still an unsurpassed obstacle. Several factors influence the efficacy of temozolomide, including the involvement of other DNA repair systems, aberrant signaling pathways, autophagy, epigenetic modifications, microRNAs, and extracellular vesicle production. The blood–brain barrier, which serves as both a physical and biochemical obstacle, the tumor microenvironment’s pro-cancerogenic and immunosuppressive nature, and tumor-specific characteristics such as volume and antigen expression, are the subject of ongoing investigation. In this review, preclinical and clinical data about temozolomide resistance acquisition and possible ways to overcome chemoresistance, or to treat gliomas without restoration of chemosensitinity, are evaluated and presented. The objective is to offer a thorough examination of the clinically significant molecular mechanisms and their intricate interrelationships, with the aim of enhancing understanding to combat resistance to TMZ more effectively.

## 1. Introduction

Glioblastoma (GB) is the most common primary malignant brain tumor in adults, comprising roughly 50% of gliomas [1]. Tumors previously called glioblastoma are, in the 2021 World Health Organization (WHO) classification, divided into two separate diagnoses based primarily on isocitrate dehydrogenase (IDH) mutation status: a. Glioblastoma, IDH-wildtype, CNS WHO grade 4; b. Astrocytoma, IDH-mutant, CNS WHO grade 4 [2]. For the purposes of this review, GB refers to IDH-wildtype glioblastoma unless specifically stated otherwise. High-grade gliomas originate from neural progenitor cells. The precise stage of differentiation of such target cells remains under investigation. Mouse models and molecular genetic studies of patient GB tissue, adjacent subventricular zone (SVZ) biopsies, and normal tissue indicate that astrocyte-like neural stem cells, which contain low-level somatic driver mutations and are in the SVZ, are the cells of origin. Over time, these cells may migrate and acquire additional somatic mutations, ultimately leading to the development of IDH-wildtype glioblastoma in distant brain regions. These multipotent tumor stem cells may have therapeutic implications, since therapeutic agents that fail to eliminate the tumor stem cells will be ineffective in eradicating the tumor. *PTEN* inactivation, *TERT* promoter mutation *EGFR*, *PDGFR*, or *MET* amplification, or in rare cases an oncogenic chromosomal translocation fusing the tyrosine kinase coding domains of *FGFR1* or *FGFR3* to transforming acidic coiled-coil 1 or 3 (*TACC1/3*), are associated with GB formation. The wingless (WNT) signaling pathway may also be aberrant in glioblastoma [3,4].

Histopathologic hallmarks include increased cellularity, nuclear pleomorphism, frequent mitoses, necrosis, and neovascularization. The presence of either TERT promoter mutation, EGFR gene amplification, or combined gain of entire chromosome 7 and loss of entire chromosome 10 (17/10) allows for a molecular diagnosis of glioblastoma, IDH wild-type, even in the absence of the characteristic GB histologic features [2,5].

Even with maximal therapy, glioblastoma has a less favorable prognosis and is less responsive to chemotherapy. The 5-year survival rate is less than 7%, with a median overall survival (mOS) of approximately 12 to 15 months with standard treatment [6]. Resistance to temozolomide (TMZ) therapy is a major cause of treatment failure. Therefore, overcoming TMZ resistance can be critical to improving treatment outcomes [1,7,8]. 

**Figure 1:**
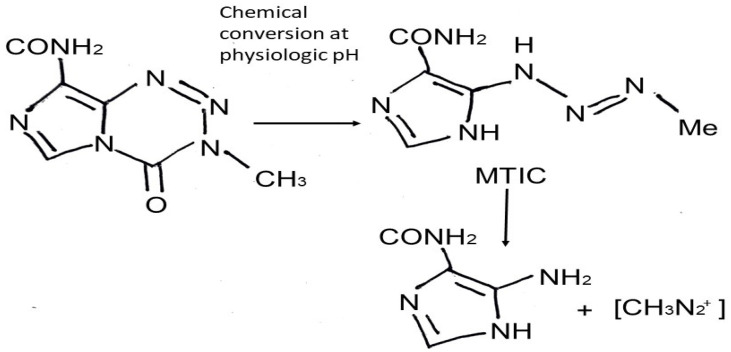
Chemical structure of temozolomide and mechanism of action (MTIC: monomethyl 5-triazino imidazole carboxamide).

TMZ is an imidazotetrazine lipophilic prodrug [9] which is rapidly and nonenzymatically converted to the active alkylating metabolite MTIC [(methyl-triazene-1-yl)-imidazole-4-carboxamide]; this conversion occurs under physiologic pH in all tissues to which it distributes [9,10] (Figure 1). Pharmacologically, after per os administration, TMZ takes approximately 0.5 to 1.5 h to reach its greatest plasma concentration. In its majority, it remains unbounded in the central circulation and demonstrates high bioavailability [11]. The cytotoxic effects of MTIC are manifested through alkylation (methylation) of DNA at the O^6^, N^7^ guanine positions, which leads to depletion of the DNA-repair enzyme O6-methylguanine-DNA methyltransferase (MGMT), DNA double strand breaks, and apoptosis [10]. DNA repair mechanisms, such as MLH1, MSH2, MSH6, and PMS2, recognize the DNA damage caused by TMZ, leading to cell cycle arrest and cells’ death [12]. Gliomas present a more alkaline environment compared to surrounding healthy brain cells, leading to drug activation [1,3,4,7,8] almost exclusively in the tumor cells [1,7,8,13,14]. Moreover, senescence induced by TMZ is another phenomenon that should also be mentioned. TMZ senescent GB cells overexpress NF-kB and lead to inflammatory cytokines’ production, such as IL6 and IL8; thus, these cells seem not to be eliminated after TMZ and radiotherapy and tend to be present even in recurrence of the tumor, with a not yet fully clarified role [15]. 

Due to the highly heterogeneous and mutation-prone nature of GB, resistance to TMZ is developed and accounts for over 50% of GB patients that eventually fail to respond to the therapy [16]. The demethylating enzyme O6-methylguanine-DNA methyltransferase (MGMT) has been implicated in intrinsic TMZ resistance (TMZ-R) and recurrence by removing alkyl groups from the O^6^ position of guanine directly [17]. Herein, we present the most recent preclinical and clinical data about TMZ resistance acquisition and possible ways to overcome this resistance. This review aims to help physicians choose the best evidence-based therapy for chemoresistant glioblastomas based on the existing literature. 

## 2. Preclinical Data

Preclinical data evaluating possible mechanisms of overcoming temozolomide resistance are presented below; a summary is presented in Supplementary Materials Tables S1–S6.

**A.**  **Targeting DNA repair mechanisms**

**MGMT**

The most common mechanism of TMZ resistance has been associated with MGMT DNA repair enzyme. This enzyme removes the methyl groups attached to O6 guanine position, correcting the lesions formed by TMZ. Molecules that inhibit MGMT, such as O6-Benzyl guanine (O6-BG) and O6-(4-bromothenyl) guanine, have been tested in animal models and in vitro, enhancing TMZ action, but their pronounced bone marrow toxicity challenges their integration into clinical practice [1,7,18,19].

**MMR mutations**

In some cases, TMZ-related DNA products can be combined with thymine, allowing the cell cycle to continue. The DNA mismatch repair (MMR) system removes thymine, arresting tumor growth. Mutations in MMR genes may lead to loss of apoptosis and cell regeneration, lowering the effectiveness of TMZ. Hunter et al. (2006) noticed MSH6 mutations in recurrent GB cell lines which were not mutated before TMZ treatment, demonstrating a potential role in TMZ resistance acquirement [20]. Li et al. (2022) proved that concomitant administration of TMZ, NAD+ precursor dihydronicotinamide riboside (NRH), and a poly(ADP-ribose) glycol hydrolase inhibitor resulted in poly(ADP-ribose) hyperaccumulation and DNA repair mechanisms such as MMR and BER knockdown, leading to an increase in TMZ cytotoxicity and effectiveness [1,7,19,21,22,23,24,25].

**BER**

The base excision repair (BER) system repairs damage caused by oxidizing agents, radiation, and alkylating factors, including N3 and N7 methylation caused by TMZ. BER component inhibitors (for example, PARP inhibitors) may be effective; however, BER’s significance in TMZ resistance is still not clear. Zampieri et al. (2021) proved that upregulation of oxidative activity in tumor cells’ mitochondria is a main cause of TMZ resistance and suppression of oxidative phosphorylation or autophagy may promote chemosensitivity [26]; olaparib, unexpectedly, seems to inhibit mitochondrial complex I, suggesting that its use should be further tested in advanced TMZ-resistant GBs [1,7,23,25,26,27].

**B.**  **Survival and metastasis regulation proteins (Galectin-1, ID1)**

**Galectin**

Galectin-1 is a member of the lectin protein family, with both intracellular and extracellular functions. It is related to tumor cell migration, formation of metastases, T-cell apoptosis, and chemotherapy and radiotherapy resistance. Davanat, a galactomannan which binds to galectin-1 at carbohydrate recognition domain, has been approved for colorectal cancer; it is not yet evaluated in GB in clinical trials [1,28].

**The role of ID1**

The inhibitors of DNA binding proteins (ID) are a family of proteins that promote cells’ survival and homeostasis. More specifically, ID1 has been found to contribute in plenty of cancer’s hallmarks, such as angiogenesis, migration, and survival of malignant cells, and its overexpression leads to EGFR dysregulation, resulting in the development of TMZ resistance. Sachdeva et al. (2019) managed to enhance sensitivity to TMZ with the concomitant use of pimozide, an FDA-approved dopamine receptor antagonist, in vitro. Pimozide seems to inhibit pEGFR and USP1, which stabilizes—via deubiquitination—the ID1 protein, and has proved its efficacy against multiple tumor pathways at in vivo mouse models too, underlying a new potential drug combination against TMZ-resistant GBs [29,30].

**Figure 2:**
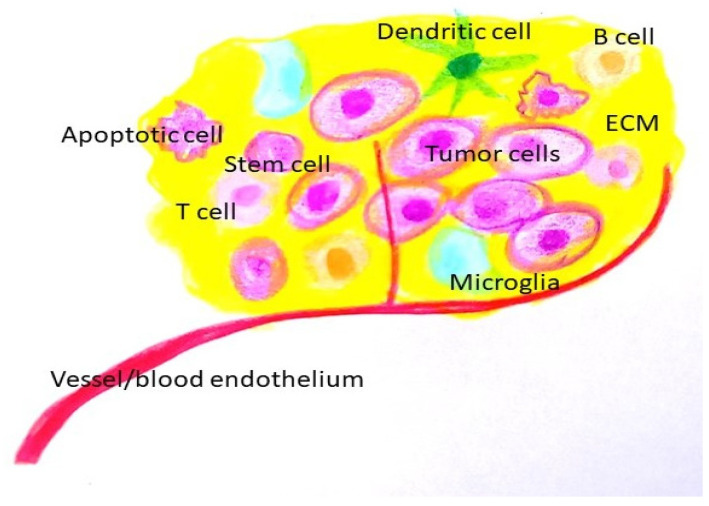
Tumor microenvironment. (ECM: extracellular matrix).

**C.**  **The role of the tumor microenvironment (TME) (Figure 2)**

The tumor microenvironment (TME) is comprised of a cellular component (glioma cells, immune cells), and non-cellular components (extracellular vesicles, extracellular matrix (ECM) components, and secreted ECM remodeling enzymes). Due to its diverse components and dynamic nature, the TME plays a vital role in the survival of cancer cells and their response to therapy.

**Targeting the cellular component of TME**
**Endothelial cells and blood–brain barrier (BBB)**

The tumor’s endothelial cells are resistant to chemotherapy, while possessing a high rate of migration and growth factor expression, and a lower proliferation rate. Tumor endothelial cells express VEGF receptors, creating a paracrine loop wherein tumor cells stimulate vascular proliferation. In GB, the microenvironment plays a crucial role, with areas of neovascularization frequently encircling necrotic zones. Additionally, other proangiogenic signal transduction pathways are heightened, including the PDGF pathway involving PDGFR-beta on endothelial cells, angiopoietin-2 and its receptor Tie-2, the fibroblast growth factor family and its receptors, and stromal-cell derived factor-one alpha. In addition, the blood–brain barrier (BBB) is made up of microvascular endothelial cells separating blood from brain interstitial fluid, while communicating with astrocytes and pericytes [31]. In GB, some areas of a tumor have BBB breakdown and increased permeability, whereas others do not. The invasive perimeters of GB consist of normal brain tissue infiltrated by cancer cells. Within this area, the BBB remains intact, limiting the delivery of drugs to these invasive margins, which harbor a distinct immune microenvironment and are the primary sites for tumor recurrences following treatment [13,32].

**Astrocytes**
**Astrocyte-induced chemoresistance**

Astrocytes are the most multitudinous glial cell population, occupying approximately 50% of the human brain’s volume. They often communicate through gap junctions, promoting astrocyte–astrocyte and astrocyte–glioma cell intercommunication, contributing to malignant cell invasion by mediating microRNA signaling. It is also believed that astrocyte-mediated transfer of protective mitochondria is involved in TMZ chemoresistance; thus, this hypothesis needs to be further verified [13,32].

**Astrocytes and stem cancer cells**

Overexpression of some transcription factors, such as Nanog, in p53-negative astrocytes, has been linked to dedifferentiation to cancer progenitor cells or cancer stem cells, retaining a possible pathway to the TMZ resistance. Glioblastoma stem cells (GSCs) possess a crucial role in TMZ resistance development due to their differentiation capacity and induction of tumor heterogeneity [23]. New therapeutic approaches targeting specific GSCs markers have emerged; CD133 is one of the most commonly used cell surface markers for GSCs, and targeted therapies towards CD133-positive cells have been developed, such as chimeric antigen receptor T (CAR T) cell therapy [23]. Moreover, autophagic cell death of CD133+ cells may become activated after sonic hedgehog signaling inhibition, through mTOR-independent pathways [33]. In previous research, the Notch pathway seemed to be upregulated in GSCs, and γ-secretase inhibitors inhibited both Notch and VEGF signaling pathways, highlighting their potential usage in TMZ-resistant GBs [34]. Lastly, GSCs are located in niches whose viability depends on their abnormal vascularity; antiangiogenic agents are thought to play a critical role in GSC survival and distribution to TMZ resistance [35,36].

**Microglia/Macrophages**

Microglia consists of CNS macrophages. Tumor-associated macrophages (TAMs) release plenty of factors that seem to contribute to glioma cell proliferation, survival, and migration. Microglia synthesizes and releases stress-inducible protein 1 (STI1), endothelial growth factor (EGF), colony stimulating factor 1 (CSF-1), CCL2 receptor (CCL2, also known as monocyte chemoattractant protein-1, is expressed by GB cells), TGF-β, membrane type 1–matrix metalloproteinase (MT1–MMP), and signaling through Toll-like receptors, which seem to retain the most critical role among others [32,37,38]. Bowman and Joyce (2014) demonstrated in preclinical stages that the use of CSF-1 inhibitors, along with TMZ and radiotherapy, may improve TMZ-resistant GB prognosis [39].

**Targeting non-cellular components of TME**

**a.**  **The role of extracellular matrix and interstitial fluid pressure**

The extracellular matrix (ECM) and interstitial fluid pressure play a role in drug distribution, from noncellular factors to the glioma cells, possessing a possible role in chemoresistance. A great number of the ECM’s components conduce to malignant cell survival through a variety of molecules: tenascin-C (TN-C), a member of the tenascin glycoprotein family, fibronectin, an ECM glycoprotein which conduces to p53 suppression, fibulin 3, another glycoprotein acting through Notch and NF-kB signaling pathways, and hyaluronic acid, a high-molecular-weight glycosaminoglycan; all of them lead to GB cell proliferation, survival, neovascularization, and chemoresistance [40,41].

**b.**  **The role of extracellular vesicles**

Extracellular vesicles (EVs) are a family of cell-derived structures possessing a role in cell-to-cell communication. They may become involved in plenty of pathological conditions and they can even be used as potential therapeutic approaches. EVs help tumor cells to communicate and secrete significant molecules for glioma cells’ survival [42,43]. 

Nandhu et al. (2018) were the first to develop an anti-fibulin 3 monoclonal antibody, giving promising results, while inducing cancer cell apoptosis and inflammatory cell proliferation of the tumor cells [44]. Radioimmunotherapy targeting the extra domain B of fibronectin [45], antibodies against tenascin-C [46], double-specific tenascin-C and fibronectin targeted peptide [47], double-stranded RNA with a nucleotide sequence homologous to tenascin-C mRNA [48], oncolytic herpes simplex virus (oHSV) armed with MMP-9 [49], HSP47 by modifying the TGF-β pathway [50], chondroitinase, riving chondroitin sulfate disaccharide chains from chondroitin sulfate proteoglycans in the tumor’s ECM [51], lumefantrine, through inhibition of the heat shock proteins-MMPs circuit [52], and tetraarsenic oxide, via decreasing MMPs and protein kinase B phosphorylation [53], seem to carve the path of new targeted therapies against advanced, chemoresistant GBs.

**D.**  **Key molecular pathways in temozolomide resistance**

**1.**  **The Akt pathway**

The Akt/mTOR (Ak strain transforming/mammalian target of rapamycin) signaling pathway possesses a crucial role in TMZ resistance acquisition, mainly by promoting tumor cell upregulation, proliferation, survival, and apoptosis blockage [54].

**2.**  **The Wnt/β-catenin pathway**

The Wnt (Wingless-related integration site)/β-catenin pathway seems to be involved in autophagy-related protein 9B activation, mainly through loss of DOC-2/DAB2-interacting protein (DAB2IP), which is a tumor suppression gene. Genome-wide binding profiles for ASCL1 and the Wnt effector LEF-1 provide mechanistic insight and suggest widespread interactions between the TF module and the signaling pathway. Regulatory connections between ASCL1, Wnt signaling, and collaborating TFs seem to be essential for the maintenance and tumorigenicity of GB cancer stem cells. Blockage of this pathway inhibits TMZ-associated autophagy and sensitizes glioma cells to chemotherapy [55].

**3.**  **The JAK/STAT pathway**

The Janus kinase/signal transducer and activator of transcription (JAK/STAT) pathway is a very complex signaling pathway; its upregulation induces angiogenesis, tumor cell proliferation, and immune blockage and suppression [56]. Kohsaka et al. demonstrated that STAT3 inhibits the degradation of the enzyme MGMT, whose expression is associated with TMZ resistance. In line with that, STAT3 inhibition and radiation in a syngeneic immune-competent glioma mouse model led to immunologic TME reprogramming, with increased dendritic cell–T-cell interaction and antigen presentation. This was correlated with significantly longer animal survival, indicating that a fully functional immune response was required to mediate the therapeutic effects of STAT3 inhibition [50].

**E.**  **Cancer cell metabolism and pH regulation**

**The role of hypoxia, pH, and glucose**

Hypoxia has many roles, including a modulation in phenotype and increasing the migration of glioma cells. Chronic hypoxia may contribute to chemoresistance due to a lack of oxygen, which is necessary for chemotherapeutic drugs to act. Moreover, cycling hypoxia has been proven to increase the delivery of ATP-binding cassette subfamily B member 1 (ABCB1), leading to resistance to TMZ as well as the deliverance of Livin, a member of apoptosis proteins, which is overexpressed in both chronic and cycling hypoxia [13,32,57,58].

PH becomes minimized close to the tumor, inhibiting gap junctions, increasing vascular endothelial growth factor (VEGF), carbonic anhydrase, interleukin-8, cathepsin B, and matrix metalloproteinase-2 and -9 expression, leading to progression, survival of the tumor cells, and chemoresistance [13,32,59].

**RFP**

RET finger protein (RFP) binds to deacetylase 1 in GBs, inducing deacetylation of H3K27 and dysregulation of cis-regulatory elements, leading to resistance in chemotherapy. Knockdown of RFP decreases oxidative stress and modifies the function of BER and, in addition to TMZ, transcends chemoresistance [60,61].

**F.**  **Molecules contributing to temozolomide resistance**

**The role of miRNAs**

MicroRNAs (miRs) are non-coding molecules that negatively affect gene expression by binding to the 3′-untranslated region of messenger RNAs [7]. A suppression of miR-519a has been connected with GB chemoresistance, leading to the collection of more preclinical data about this hypothesis [49]. In vivo and in vitro analysis has demonstrated that miR-519a is able to inhibit the STAT3/Bcl-2/Beclin-1 pathway, giving rise to GB cell apoptosis through autophagy, sensitizing gliomas to TMZ treatment. Cardoso et al. highlighted that overexpression of miR-200c downregulates GB cells’ metabolism, promotes chemosensitivity, and leads to cancer cell cycle arrest [62,63].

**AURKB**

Aurora kinase B (AURKB) is a member of the aurora kinases, a family of serine/threonine kinases that contribute to chromatid separation. Moreover, AURKB is already recognized as a possible encoding gene possessing a critical role in TMZ resistance acquisition, glioma cell survival, proliferation, apoptosis, and autophagy, by becoming involved in p53/Mdm2 suppression, PI3K/Akt/mTOR, p38 MAPK and AMPK signaling pathway alternation. Its inhibition has been tested both in vitro and in vivo, through transfection via lentivirus and specific small molecules targeted against AURKB, such as Hesperadin. These agents, as monotherapy or concomitant to TMZ (for Hesperadin), led to TMZ sensitivity, and their efficacy should be further examined in clinical trials [54,64,65].

**De novo purine synthesis**

Shireman et al. highlighted the importance of ADP-ribosylation factor-like protein 13B (ARL13B), a ciliary protein whose epigenetic modification may possess a crucial role in the development of chemoresistance in GBs. It was unveiled that its interaction with inosine-50-monophosphate dehydrogenase 2 (IMPDH2), a purine biosynthetic enzyme, reduces the DNA-associated damage caused by TMZ, which downregulates purine salvage; in vivo use of the FDA-approved drug mycophenolate mofetil, which can block IMPDH2’s activity and diminish the efficiency of the ARL13B–IMPDH2 circuit, led to enhancement of TMZ therapeutic efficacy [66]. In addition, Zhou et al. (2020) demonstrated that high activity of GTP synthetase resulted in shortening of overall survival in patients with advanced GBs, and its inhibition may be an auspicious strategy [67].

**NF-kB**

The NF-kB family of transcriptional factors controls the expression of plenty of targeted genes that are involved in cell growth, survival, apoptosis, et cetera [68]. Yu et al. demonstrated that high NF-kB activity is associated with oncogenic characteristics of glioma cells that promote GB’s viability; moreover, inhibition of NF-kB resulted in S-cell cycle arrest and apoptosis. Furthermore, NF-kB correlates with MGMT expression in gliomas; the use of NF-kB inhibitors, such as parthenolide, leads to MGMT downregulation, resulting in TMZ chemosensitivity restoration [69]. Moreover, Avci et al. (2020) tested in vivo the concurrent administration of an NF-κB inhibitor, BAY 11-7082, with TMZ, which resulted in contiguous outcomes, possibly by modulating the actin cytoskeleton pathway [70].

**LGR6**

Leucine-rich repeat-containing G-protein-coupled receptor 6 (LGR6) seems to promote cell proliferation and survival in plenty of malignancies, including, for example, colorectal cancer. Cheng et al. proved that LRG6 induces GB survival and chemoresistance, partly by activating the Akt signaling pathway, pointing out the potential benefit from such pathway inhibitors [71].

**Hexokinase 2**

Zhang et al. revealed that hexokinase 2 (HK2)-mediated glycolysis is of great importance in TMZ resistance emergence in GBs, resulting in malignant cell proliferation and survival. HOTAIR, a lncRNA, regulates HK2 expression in tumor cells, inhibiting miR-125. This results in HOTAIR shut down and downregulates HK2 expression, inducing chemosensitivity both in vitro and in vivo [72].

**Circular RNA ASAP1 and NRAS/MEK1/ERK 1-2 pathway**

Circular RNAs (circRNAs) are members of the non-coding RNA family, derived from RNA backsplicing and forming a covalent closed-loop structure. More precisely, circular RNA ADP-ribosylation factor GTPase activating proteins with the Src homology 3 domain, ankyrin repeat and Pleckstrin homology domain 1 (circASAP1) were found highly expressed in recurrent GBs. Wei et al. revealed that eukaryotic translation initiation factor 4A3 (EIF4A3) bounds to the 3’ flanking region of circASAP1, winding up its expression. CircRNA’s sponging to miR-502-5p eventually activated NRAS/MEK1/ERK 1-2 signaling pathway, contributing to TMZ resistance by promoting GB cell growth and apoptosis suppression [73].

**PTRF/Cavin-1**

Polymerase I and transcript release factor (PTRF), cavin-1, was situated to be involved in extracellular vesicle (EV) formation and excretion emerging from eukaryotic cells; these EVs appear to possess an essential role in GB cells’ communication, by transmitting regulatory RNA, DNA, and proteins. GB cells seem to overexpress PTRF, leading to TMZ resistance through extracellular vehicles [74]. Chloroquine downregulates PTRF expression—with still not completely understood mechanisms—and, sequentially to TMZ chemotherapy, it seems to increase TMZ’s intracellular concentration and sensitivity [75].

**Cytosolic phospholipase A2 alpha**

Yang et al. demonstrated that cytosolic phospholipase A2 alpha (cPLA2a), an intracellular enzyme that delivers arachidonic acid for eicosanoid production, induces TMZ resistance when upregulated, due to PI3K/Akt/mTOR signaling pathway overactivation. CPLA2a inhibition, and PI3K’s by extension, restore chemosensitivity in advanced, TMZ-resistant GBs [76].

**Long noncoding RNA**

Long noncoding RNA (lncRNA) transmission via EVs from one cell, cancerous or not, to another may affect a tumor’s expansion and progression [77]. Li et al. revealed through their study that EV-derived TMZ-associated lncRNA in GB (lnc-TALC) is able to promote p38 MAPK pathway activation, inducing C5a release, via microglia M2 macrophage polarization through enolase 1 (ENO1) binding, leading to TMZ resistance. Therefore, this study enlightens the pathway of finding novel therapeutic agents, by blocking the lnc-TALC-mediated communication between GB cells and microglia [78].

## 3. Clinical Data

One way to ameliorate our therapeutic approach to GB is either to replace temozolomide with a targeted agent to improve the toxicity profile with the same efficacy, or to add a new agent with non-cross toxicities to increase treatment efficacy and overcome acquired resistance. Supplementary Material Table S7 summarizes the clinical data that are discussed below.

### 3.1. Bevacizumab and Other Antiangiogenic Agents

GB is one of the most vascularized human malignancies due to its wide production of angiogenic factors, such as VEGF. It is proved that VEGF not only induces neovascularization in GBs, but also modifies BBB’s vascular permeability and generates vasogenic edema; its inhibition prevents glioma cells’ growth, proliferation, and angiogenesis [32,75,76]. Bevacizumab is the most thoroughly examined agent in patients with GBs, with a number of clinical trials being completed or recruiting at the moment, testing its efficacy as monotherapy or in combination with standard chemotherapy, mainly irinotecan [77,78,79,80,81,82,83,84]. Different combinatorial approaches are currently tested in clinical trials such as of bevacizumab along with marizomib (NCT02330562), bevacizumab, and irinotecan (BI) [85], and TMZ plus apatinib (NCT03741244). In general, bevacizumab has been shown to increase progression-free survival (PFS) but not overall survival (OS) in patients with firstly recurrent glioblastoma [86].

### 3.2. EGFR Inhibitors

Endothelial growth factor receptor (EGFR) is a receptor which is of great importance in migration, proliferation and angiogenesis of malignant cells. Its mutations are encountered at 40% of gliomas, resulting in the production of targeted therapies, such as monoclonal antibodies and tyrosine kinase inhibitors [1,79,80,81]. The usage of Cetuximab in TMZ-resistant GBs was disappointing in clinical trials (interruption at phase II), while the use of Erlotinib and Gefitinib (and other TKIs, such as Afatinib; Phase II NCT00727506) was found to be effective in a subpopulation of patients with intact PTEN and MGMT promoter methylation [1] (NCT00052208, NCT00525525, NCT00445588).

### 3.3. The PI3K Pathway

The importance of the PI3K pathway’s dysregulation has been highlighted in the development of multiple cancer types. The PI3K family of intracellular kinases possesses a critical role in cell proliferation, survival, migration, and differentiation. PI3K/AKT/mTOR and some of the involved proteins, such as p85, PTEN, EGFR, are often negatively alternated in GB [82]. Moreover, BKM120, a pan-PI3K inhibitor, is investigated in concomitant use with bevacizumab in a phase I/II clinical trial, resulting in a median progression-free survival of 4.0 months, a 26% overall response rate, and a progression-free survival at 6 months at 36.5%, demonstrating no superiority to isolated bevacizumab usage [83].

### 3.4. TGF-β Inhibitors

Tumor growth factor-β (TGF-β) is a multifaceted cytokine, whose signaling pathway contributes to cell proliferation, survival, and apoptosis in plenty of cell series, including GB cells [84]. It activates plenty of intracellular pathways and is associated with the knockdown of the SUMO gene, which seems to contribute to the development of TMZ resistance in gliomas. Concomitant treatment with TMZ and OKN-007, a TGF-β inhibitor, was found to be superior to monotherapy with TMZ in preclinical series [85]. Bogdan et al. designed a phase IIb clinical trial, evaluating the effectiveness and safety of trabedersen (AP 12009), a TGF-β2 inhibitor, compared with standard chemotherapy for high-grade gliomas; median survival was 39.1 months for 10 µM trabedersen, 35.2 months for 80 µM, and 21.7 months for standard chemotherapy group. The results remain not statistically significant [86].

### 3.5. Antibody Drug Conjugates

Antibody drug conjugates (ADCs) consist of a monoclonal antibody specific for a certain cell surface antigen attached to a cytotoxic agent. Their efficacy in plenty of cancer types is undebatable, as possessing lower possibility of complications and a tumor-specific anti-oncogenic effect; however, their efficiency and safety in advanced, TMZ-resistant GBs remains controversial [16,87]. A large number of ADCs has been tested; 131I radio-conjugated antibodies [88], 131I-labeled murine antitenascin monoclonal antibody 81C6 (131I-81C)] [89], 125I-labeled anti-epidermal growth factor receptor 425 murine monoclonal antibody (125I-mAb 425) [90], ligand-targeted toxin conjugate Transferrin-CRM107 (Tf-CRM107) [91], EGFR targeting recombinant toxin (TP-38) [92], convection-enhanced delivery of IL13-PE38QQR versus Gliadel [93], and IL-4 Pseudomonas exotoxin (NBI-3001) [94]. Contrarily, the usage of more recent ADCs is remarkably promising; ABT-414, depatuxizumab mafodotin, an EGFR targeting ADC [95,96] (p4) and AMG 595 [97], an anti-EGFRvIII ADC, remain the most promising potential therapeutic approaches.

### 3.6. NTRK Inhibitors

Neurotrophic tyrosine receptor kinase (NTRK) fusion mutations are detected in approximately <2% of GBs; however, it is known that these mutations lead to Tropomyosin receptor kinase (Trk) activation and tumorigenesis, possessing the possible role of a drive mutation in some types of GBs [98]. The role of repotrectinib (NCT04094610), like the role of larotrectinib (NCT02637687, NCT02576431) is being studied in phase I/II trials at the moment. Entrectinib, an NTRK/ROS1 (c-ros oncogene 1)/ALK (anaplastic lymphoma kinase) pathway inhibitor, was tested, with promising results, in a case of a radiation recurrent GB [99].

### 3.7. BRAF Inhibitors

V-raf murine sarcoma viral oncogene homolog B1 (BRAF) is a serine/threonine kinase protein that signals the Ras-Raf-MEK pathway and is mutated in 1–8% cases of GBs [100]. Dabrafenib alone [101] or combined with trametinib in patients with BRAF V600E-mutant low-grade and high-grade glioma showed beneficial results in a ROAR study [102], while vemurafenib had mixed results that varied according to the histological subtype of the tumor [103]. Clinical trials are running at the moment to test the efficacy of dabrafenib and trametinib co-administration in high-grade brain tumors, including GBs (NCT03593993, NCT03919071), while one study examines the use of binimetinib with encorafenib (NCT03973918).

### 3.8. Bevacizumab-Modified Docetaxel-Loaded Nanostructured Lipid Carriers

Docetaxel is a chemotherapeutic agent with significant anti-tumor legacies; however, its clinical usage in GB is limited due to its sparing brain distribution through BBB. Di Filippo et al. (2022) manufactured an agent that could cross BBB and precisely release docetaxel only at GB cells, via docetaxel-loaded nanostructured lipid carriers attached to bevacizumab (BVZ-NLC-DTX). They managed to prove that BVZ-NLC-DTX led to malignant cells’ apoptosis, via targeting specifically VEGF overexpressing cells, and leaving intact the surrounding, healthy brain parenchyma. Its efficacy in vitro and in vivo remains to be reproduced in future clinical trials [104].

### 3.9. Immunotherapy

Immunotherapy retains a critical position in therapeutic novelties of plenty of cancer types. Its clinical usage in GBs is, nonetheless, not thoroughly examined [105].

### 3.10. Immune Checkpoint Inhibitors (ICPIs)

ICPIs on GB, such as Nivolumab (an anti-Programmed Cell Death Protein 1 PD-1 inhibitor/anti-PD 1 inhibitor) with or without Ipilimumab (an anti- cytotoxic T-lymphocyte-associated antigen 4 inhibitor/an anti-CTLA-4 inhibitor) or TMZ plus radiotherapy, is tested in current clinical trials (NCT02017717, NCT02667587). Uprising ICPIs, like magrolimab, an anti-CD47, and other anti-CD73 monoclonal antibodies, are investigated in phase II (NCT02953509, for hematological malignancies) and phase I clinical trials, respectively (NCT02503774 for a variety of solid tumors).

### 3.11. Chimeric Antigen Receptor T (CAR-T)

CAR-T cell therapy has been examined in a first-in-human administration study with 10 recurrent GB patients enrolled; nevertheless, heterogeneity and TME’s vital contribution to malignant cell survival are yet to be overcome [106].

### 3.12. The Role of Sorafenib

Sorafenib is a versatile kinase inhibitor capable of blocking multiple kinases, such as VEGFR-2, VEGFR-3 (Vascular Endothelial Growth Factor), c-RAF (proto-oncogene Rapidly Accelerated Fibrosarcoma), RET (Rearranged during transfection), wildtype and V599E mutant B-Raf (V-Raf Murine Sarcoma Viral Oncogene Homolog B), fibroblast growth factor receptor 1, p38α, platelet-derived growth factor receptor β (PDGFRβ), c-KIT and Flt3, resulting in both tumor cell suppression and inhibition of GB’s neovascularization [107]. Sorafenib’s usage as first-line therapy ended up disappointing [108], like its coordinating administration with erlotinib, an EGFR inhibitor [109].

### 3.13. IDH Mutations

Isocitrate dehydrogenase (IDH) mutations are most common in grade 2 and 3 gliomas but can also be seen in tumors with grade 4 histology; the latter are no longer referred to as IDH-mutated glioblastoma according to the 2021 WHO classification of CNS tumors and are now called “astrocytoma, IDH mutated, WHO grade 4”. Regardless of grade, IDH mutations in glioma are associated with younger age and comparatively favorable prognosis [2], while olutasidenib (FT-2102), an IDH1 inhibitor, was examined in a phase Ib/II clinical trial, giving a 12-month PFS rate of 20.8% in patients with relapsing glioma [110]. Vorasidenib (AG-881) has shown preliminary antitumor activity in patients with recurrent or progressive nonenhancing mIDH lower-grade gliomas (NCT02481154) [111], whereas in patients with mIDH1 advanced glioma, ivosidenib (AG-120) was associated with a favorable safety profile, prolonged disease control, and reduced growth of nonenhancing tumor (NCT02073994). The dual IDH1/IDH2 inhibitor vorasidenib exhibited better brain permeability and target engagement than ivosidenib in a pilot perioperative randomized clinical trial in patients with IDH1-mutant glioma (NCT03343197) [112]. Other agents such as BAY1436032 are also under investigation at the moment (NCT02746081).

### 3.14. Other Therapies

p53 is a protein produced by a tumor suppressor gene, TP53, while Mdm2 gene overexpression leads to p53 suppression. Nutlin-3 is a molecule that inhibits p53-Mdm2 binding, with interesting results in the preclinical stage [1,113]. Adenoviral-associated gene therapy for p53 has shown improvement in quality of life and optimization of prognosis in patients with recurrent GBs in some clinical trials [1]. Moreover, Heimberger et al. are running a clinical trial, which started back in 2013 and is at phase I at the moment with a STAT3 inhibitor, WP1066, for newly diagnosed or recurrent GB (*NCT01904123*). An interventional, non-randomized clinical trial (phase Ib/II) with napabucasin (BBI608), a STAT3 inhibitor, is being conducted comparing the use of napabucasin with TMZ versus standard therapy for recurrent or progressed glioma (*NCT02315534*). Sotelo et al. are investigating the use of chloroquine as adjuvant therapy to GB in an interventional, double-blinded, phase III clinical trial (*NCT00224978*). Additionally, a phase II trial tested the use of regorafenib, a multi-kinase inhibitor (VEGFR1-3, KIT, RET, BRAF, and others), resulting in an increase in OS rate in recurrent GBs. Its promising results indicate that its administration should be examined in a phase II clinical trial [114]. Furthermore, gamma-secretase inhibitors (GSIs) inhibit the Notch pathway and have been tested in clinical trials in concomitant use with TMZ, giving fruitful results [115].

### 3.15. Neuronavigation-Guided Focused Ultrasound

The BBB, the basal vascular structure of the brain, is composed of endothelial cells, pericytes, and astrocytes’ end-feet, restricting more than 95% of drug’s supply to the brain parenchyma. Chen et al. planned a phase I clinical trial, testing the use of neuronavigation-guided focused ultrasound (NaviFUS) to modify the BBB and potentially improve drug delivery in recurrent GBs, with less significant adverse effects. A phase II clinical trial is under construction [116].

### 3.16. Tumor-Treating Fields/Synergy with Temozolamide

A promising therapy in the field of oncology is tumor-treating field therapy (TTF-T), with increasing usage in a variety of tumor types. TTF-T, via impeding the transition from metaphase to anaphase, has gained popularity among novel therapeutic treatments in variable malignancies, including GBs, demonstrating their effectiveness in preclinical stages [117]. Stupp et al. (2012) carried out a phase III clinical trial comparing TTF-T to standard chemotherapy; this study did not reach its primary end-points, but displayed similar efficacy between TTF-T and chemotherapy, along with more favorable toxicity and quality of life for the TTF group [118]. Five years later, Stupp et al. (2017) managed to demonstrate that patients who received TTF-T combined with TMZ, versus TMZ monotherapy, achieved statistically significant although modest prolongation in progression-free survival and overall survival [119].

### 3.17. Photodynamic Therapy

Photodynamic therapy is an emerging therapy for plenty of cancer types, including glioblastomas. It is a light-based therapy which delivers the drug specifically to the tumor site, reducing drug toxicity to normal, healthy surrounding tissues [120]. In the past, phototherapy was mainly linked to the administration of 5-aminolevulinic acid (5-ALA), which leads to the release of reactive oxygen species (ROS) and cancer cell apoptosis. Nowadays, the use of plenty of molecules along with photodynamic therapy is tested; nanoparticle-linked microRNAs as photosensitizers, near-infrared light delivery, nanoparticle-like photosensitizers, phytocompounds, etc., are the main examples [121]. Clinical trials are running at the present time, including NCT04469699, NCT03897491, and NCT04391062.

**Nanoparticle (NP)-Based Combinational Strategies for Overcoming the BBB**

Ongoing research is increasingly directed towards exploring diverse mechanisms to achieve targeted drug delivery into the brain. The use of genetically modified cells or the engineering of living cells with functionalized NP provides exciting avenues for tailored and targeted therapies. Combining nanoparticle-based drug delivery systems with cell-based carriers not only enhances the delivery of therapeutic agents but also presents additional benefits such as immunomodulation, tumor targeting, and the prospect of personalized medicine [122].

**Figure 3:**
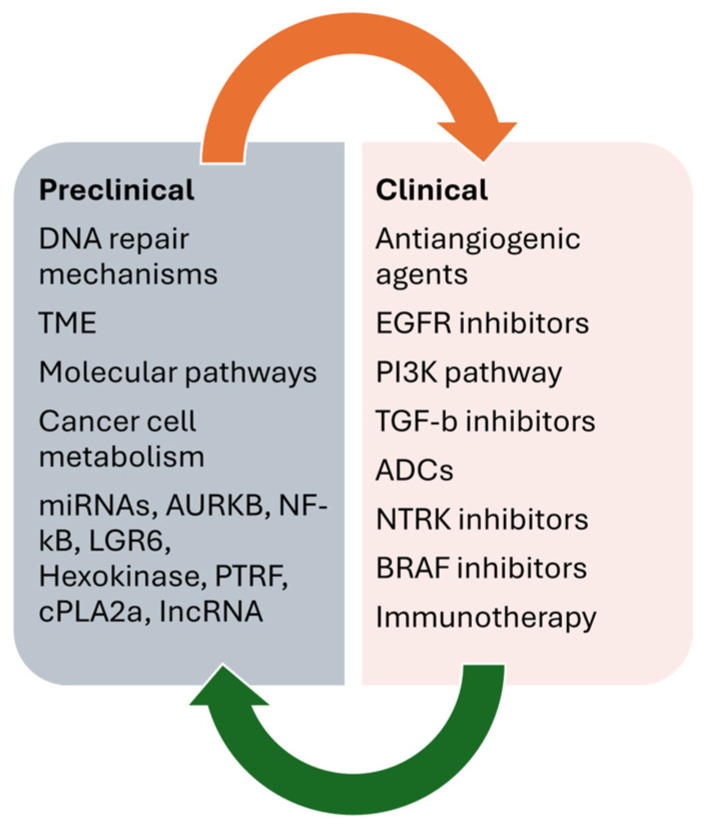
Main preclinical and clinical data on overcoming resistance to temozolomide in GB.

Figure 3 summarizes the main preclinical and clinical data on overcoming resistance to temozolomide in GB.

## 4. Future Approaches

Currently, preclinical and clinical research investigates potential mechanisms to overcome TMZ resistance in GBs.

Despite multimodal treatment including surgery, radiation therapy, and/or chemotherapy, GB IDH wild-type has a real-world median overall survival of approximately 12 to 15 months, although recent phase 3 clinical trials have suggested that the median overall survival may be closer to 18 to 20 months in selected populations with good performance status [123]. No uniform standard of care exists for the treatment of recurrent glioblastoma. Treatment options are tailored to the individual patient. A second resection may be a reasonable option for selected patients [124]. Although bevacizumab remains the only FDA-approved systemic therapy for recurrent glioblastoma, current data show that it does not improve overall survival.

TMZ resistance remains a major limitation in the treatment of GB and contributes to the dismal prognosis. Dissecting the mechanisms of TMZ resistance might impact the subsequent treatment options and guide clinical decisions. In this review, we tried to explore potential strategies to maximize therapeutic potential of novel targets, achieve better patient selection, and identify novel methods of GB management.

Regarding the underlying resistance mechanisms of TMZ, rationales have been proposed to combine TMZ with another reagent to seek for opportunity to enhance the drug efficacy or to develop novel targeted therapies by modulating selective factors related to cancer pathways, energy metabolism, and adaptation in the microenvironment. Though the inhibition may not be straightforward, the rationale suggests a potential framework to benefit cancer treatment.

Among the most promising therapeutic agents discussed, ADCs have shown to be effective and safe therapies transforming treatment paradigms in various solid tumors, including CNS. More sophisticated biomarkers to select patients are needed for these new agents, since their effectiveness is correlated with tumor target expression. Furthermore, emerging data suggest that selection based on payload sensitivity may add additional value above simple target expression. In addition, improving drug access to tumors across the BBB remains an unmet need.

## 5. Conclusions

Advanced TMZ-resistant GBs remain a challenging research domain for the scientific community, still possessing the role of the most lethal primary brain tumor, with devastating overall survival and prognosis. The mechanisms involved in the development of the TMZ resistance are steadily unraveling; however, more research needs to be performed in order to fully explain this immensely complicated phenomenon. Exploring preclinical and clinical data reveals promising avenues for future therapeutic strategies that not only target restoring sensitivity to temozolomide (TMZ), but also aim to achieve the ultimate primary endpoint: effective treatment of the disease.

## Supplementary Materials

The following supporting information can be downloaded at: https://www.mdpi.com/article/10.3390/life14060673/s1. Table S1. Preclinical data/Targeting DNA repair mechanisms. Table S2. Preclinical data/Survival and metastasis regulation proteins (Galectin-1, ID1). Table S3. Preclinical data/TME. Table S4. Preclinical data/Key molecular pathways. Table S5. Preclinical data/Cancer cell metabolism and pH regulation. Table S6. Preclinical data/Molecules contributing to temozolomide resistance. Table S7. Clinical data.
