# Supplementary material for: Overcoming Resistance to Temozolomide in Glioblastoma: A Scoping Review of Preclinical and Clinical Data
Source: Life (Basel). 2024 May 24;14(6):673. doi: 10.3390/life14060673 (PMC11204771; doi:10.3390/life14060673)
Supplement: Supplementary file 1 [file life-14-00673-s001.zip › life-2952032-supplementary.pdf]

Review

Overcoming Resistance to Temozolomide in Glioblastoma: A Scoping Review of Preclinical and Clinical Data

Dimitra Smerdi [1], Myrto Moutafi [1,*], Ioannis Kotsantis [1], Lampis C. Stavrinou [2] and Amanda Psyrri [1]

[1] Department of Medical Oncology, Second Department of Internal Medicine, "Attikon" University General Hospital, Athens Medical School, National and Kapodistrian University of Athens, 11528 Athens, Greece; demi.smerdi@gmail.com (D.S.); ikotsantis@gmail.com (I.K.); psyrri237@yahoo.com (A.P.)
[2] Department of Neurosurgery and Neurotraumatology, "Attikon" University General Hospital, Athens Medical School, National and Kapodistrian University of Athens, 12462 Athens, Greece; lampis.stavrinou@gmail.com
* Correspondence: mirtomou@gmail.com

Table S1. Preclinical data/Targeting DNA repair mechanisms

Targeting site	Resistance mechanism	Source
MGMT	removes the methyl groups attached to O6 guanine position, correcting the lesions formed by TMZ	1,7,18,19
MMR	removes thymine, arresting tumor growth	1,7,19-25
BER	repairs damages caused by oxidizing agents, radiation and alkylating factors, including N3 and N7 methylation caused by TMZ	1,7,23,25-27

Table S2. Preclinical data/ Survival and metastasis regulation proteins (Galectin-1, ID1)

Targeting site	Repair mechanism	Source
Galectin-1	member of lectin proteins◎ family, related to tumor cell migration, formation of distant lesions, T-cell apoptosis, chemotherapy and radiotherapy resistance	1,28
ID1	contributes in angiogenesis, migration and survival of malignant cells. Its overexpression leads to EGFR dysregulation, resulting in the development of TMZ resistance	29,30

Table S3. Preclinical data/ TME

Targeting site	Repair mechanism	Source
Astrocytes	associated to tumor stem cells and promotion of resistance	13,23,32-36
Microglia/Macrophages	Tumor Associated Macrophages release plenty variety of factors that seem to contribute to glioma cell proliferation, survival and migration	32,37-39
ECM	plays a role in drug distribution, from noncellular factors to the glioma cells, possessing a possible role in chemoresistance	40,41
Extracellular vesicles	cell-derived structures possesing a role in cell-to-cell communication	42-53

Table S4. Preclinical data/ Key molecular pathways

Targeting site	Repair mechanism	Source
Akt pathway	promotes tumor cell upregulation, proliferation, survival and apoptosis blockage	54
The Wnt/β-catenin pathway	loss of DOC-2/DAB2 interacting protein (DAB2IP), which is a tumor suppression gene	55
The JAK/STAT pathway	its upregulation induces angiogenesis, tumor cell proliferation, immune blockage and suppression	50,56

Table S5. Preclinical data/ Cancer cell metabolism and pH regulation

Targeting site	Repair mechanism	Source
Hypoxia, pH	contributes to chemoresistance due to the lack of oxygen, which is obligatory for chemotherapeutic drugs to act	13,32,57-59
RFP	leads to dysregulation of cis-regulatory elements, leading to resistance in chemotherapy	60-61

Table S6. Preclinical data/ Molecules contributing to temozolomide resistance

Targeting site	Repair mechanism	Source
miRNAs	negatively affect gene expression	7,49,62,63
AURKB	gets involved in p53/Mdm2 suppression, PI3K/Akt/mTOR, p38 MAPK and AMPK signaling pathway alternation	54,64,65
De novo purine synthesis	ARL13B-IMPDH2 circuit and resistance occurrence	66,67
NF-kB	provides oncogenic characteristics of glioma cells that promote GB's viability; its inhibition resulted in S cell cycle arrest and apoptosis	68-70
LGR6	potential activating of Akt pathway	71
Hexokinase-2	inhibiting miR-125, results in HOTAIR shut down and downregulates HK2 expression, inducing chemosensitivity	72
circASAP1	CircRNA's sponging to miR-502-5p eventually activated NRAS/MEK1/ERK 1-2 signaling pathway, contributing to TMZ resistance	73
PTRF	gets involved in EV formation and excretion emerged by eukaryotic cells; possess an essential role in GB cells' communication	74,75
cPLA2a	induces TMZ resistance when upregulated, due to PI3K/Akt/mTOR signaling pathway overactivation	76
Long noncoding RNA	promotes p38 MAPK activation leading to TMZ resistance, may affect tumor's expansion and progression	77,78

Table S7. Clinical data.

Group	*Description*	*Source*
Antiangiogenic factors	bevacizumab as monotherapy or combined therapy with chemotherapy or uprising agents, apatinib, marizomib	NCT02330562, NCT03741244, 32, 75-86
EGFR inhibitors	Cetuximab<br>Afatinib<br>Erlotinib/ Gefitinib	NCT00311857<br>NCT00727506<br>NCT00052208,

		NCT00525525, NCT00445588<br>1,79,81
PI3K inhibitors	XL147, Exelixis<br>XL765, Exelixis<br>BKM120, Novartis	82,83
TGF-β inhibitors	inhibit TGF-b pathway and sensitize glioma cells to TMZ	84-86
ADCs	131I radio-conjugated antibodies, 131I-81C, 125I-mAb 425, Tf-CRM107, TP-38, IL13-PE38QQR, NBI-3001, ABT-414, AMG 595	16,87-97
NTRK inhibitors	repotrectinib<br>arotrectinib<br>selitrectinib<br>crizotinib<br>merestinib<br>entrectinib	NCT04094610,NCT03093116<br>NCT02637687, NCT02576431<br>NCT03215511<br>NCT02270034<br>NCT02920996<br>98,99
BRAF inhibitors	dabrafenib and trametinib, binimetinib with encorafenib	NCT03593993,<br>NCT03919071, NCT03973918<br>100-103
Bevacizumab-modified docetaxel-loaded nanostructured lipid carriers	malignant cells◎apoptosis while leaving intact the healthy tissue	104
Immunotherapies and vaccines	immune check-point inhibitors, CAR-T cell therapy, vaccine therapy	NCT02017717,NCT02667587,<br>NCT02953509, NCT02503774<br>105,106
Sorafenib	multiple kinase inhibitor	107-109

IDH mutations	involved into many cellular pathways, such as Krebs cycle: Vorasidenib (AG-881), ivosidenib (AG-120) Olutasidenib (FT-2102),	NCT02481154<br>NCT02073994<br>NCT02746081<br>2,110-112
p53 gene therapy	adenoviral gene therapy	1,113
STAT3 inhibitors	examines the usage of WP1066 in newly diagnosed or recurrent GB (phase I)	NCT01904123
Napabucasin	compares the concomitant use of napabucasin and TMZ versus standard therapy for recurrent or progressed gliomas (phase Ib/II)	NCT02315534
Chloroquine	chloroquine as adjuvant therapy for GBs with primary endpoint malignancy's treatment	NCT00224978
Regorafenib	multi-kinase inhibitor (VEGFR1-3, KIT, RET,BRAF and others)	114
Gamma secretase inhibitors	inhibit the Notch pathway and have been tested in clinical trials in concomitant administration with TMZ	115
NaviFUS	modificates BBB's permeability and improves drug delivery in recurrent GBs	116
Tumor treating fields	imped the transition from metaphase	117-119
Phototherapy	light-based therapy which delivers the drug specifically to the tumor site	120, 121

NP-Based Combinational Strategies for Overcoming the BBB	The use of genetically modified cells or the engineering of living cells with functionalized NP provides exciting avenues for tailored and targeted therapies	122

References

1. Messaoudi, K.; Clavreul, A.; Lagarce, F. Toward an effective strategy in glioblastoma treatment. Part I: Resistance mechanisms and strategies to overcome resistance of glioblastoma to temozolomide. *Drug Discov. Today* **2015**, *20*, 899–905. https://doi.org/10.1016/j.drudis.2015.02.011.
2. Louis, D.N.; Perry, A.; Wesseling, P.; Brat, D.J.; Cree, I.A.; Figarella-Branger, D.; Hawkins, C.; Ng, H.K.; Pfister, S.M.; Reifenberger, G.; et al. The 2021 WHO Classification of Tumors of the Central Nervous System: A summary. *Neuro-Oncology* **2021**, *23*, 1231–1251. https://doi.org/10.1093/neuonc/noab106.
3. Galbraith, K.; Snuderl, M. Molecular Pathology of Gliomas. *Surg. Pathol. Clin.* **2021**, *14*, 379–386. https://doi.org/10.1016/j.path.2021.05.003.
4. Chen, R.; Cohen, A.L.; Colman, H. Targeted Therapeutics in Patients With High-Grade Gliomas: Past, Present, and Future. *Curr. Treat. Options Oncol.* **2016**, *17*, 42. https://doi.org/10.1007/s11864-016-0418-0.
5. Taal, W.; Bromberg, J.E.; van den Bent, M.J. Chemotherapy in glioma. *CNS Oncol.* **2015**, *4*, 179–192. https://doi.org/10.2217/cns.15.2.
6. Ostrom, Q.T.; Price, M.; Neff, C.; Cioffi, G.; Waite, K.A.; Kruchko, C.; Barnholtz-Sloan, J.S. CBTRUS Statistical Report: Primary Brain and Other Central Nervous System Tumors Diagnosed in the United States in 2015–2019. *Neuro-Oncology* **2022**, 24 (Suppl. 5), v1–v95. https://doi.org/10.1093/neuonc/noac202.
7. Singh, N.; Miner, A.; Hennis, L.; Mittal, S. Mechanisms of temozolomide resistance in glioblastoma-a comprehensive review. *Cancer Drug Resist.* **2021**, *4*, 17–43. https://doi.org/10.20517/cdr.2020.79.
8. Stupp, R.; Mason, W.P.; van den Bent, M.J.; Weller, M.; Fisher, B.; Taphoorn, M.J.B.; Belanger, K.; Brandes, A.A.; Marosi, C.; Bogdahn, U.; et al. Radiotherapy plus Concomitant and Adjuvant Temozolomide for Glioblastoma. *N. Engl. J. Med.* **2005**, *352*, 987–996. https://doi.org/10.1056/NEJMoa043330.
9. Marchesi, F.; Turriziani, M.; Tortorelli, G.; Avvisati, G.; Torino, F.; De Vecchis, L. Triazene compounds: Mechanism of action and related DNA repair systems. *Pharmacol. Res.* **2007**, *56*, 275–287. https://doi.org/10.1016/j.phrs.2007.08.003.
10. Villano, J.L.; Seery, T.E.; Bressler, L.R. Temozolomide in malignant gliomas: Current use and future targets. *Cancer Chemother. Pharmacol.* **2009**, *64*, 647–655. https://doi.org/10.1007/s00280-009-1050-5.
11. Karve, A.S.; Desai, J.M.; Gadgil, S.N.; Dave, N.; Wise-Draper, T.M.; Gudelsky, G.A.; Phoenix, T.N.; DasGupta, B.; Yogendran, L.; Sengupta, S.; et al. A Review of Approaches to Potentiate the Activity of Temozolomide against Glioblastoma to Overcome Resistance. *Int. J. Mol. Sci.* **2024**, *25*, 3217. https://doi.org/10.3390/ijms25063217.
12. Shaw, R.; Basu, M.; Karmakar, S.; Ghosh, M.K. MGMT in TMZ-based glioma therapy: Multifaceted insights and clinical trial perspectives. *Biochim. Biophys. Acta (BBA)-Mol. Cell Res.* **2024**, *1871*, 119673. https://doi.org/10.1016/j.bbamcr.2024.119673.
13. Zhang, X.; Ding, K.; Wang, J.; Li, X.; Zhao, P. Chemoresistance caused by the microenvironment of glioblastoma and the corresponding solutions. *Biomed. Pharmacother.* **2019**, *109*, 39–46. https://doi.org/10.1016/j.biopha.2018.10.063.
14. Zhang, J.; Stevens, M.F.; Bradshaw, T.D. Temozolomide: Mechanisms of Action, Repair and Resistance. *Curr. Mol. Pharmacol.* **2012**, *5*, 102–114. https://doi.org/10.2174/1874467211205010102.
15. Kaina, B. Temozolomide, Procarbazine and Nitrosoureas in the Therapy of Malignant Gliomas: Update of Mechanisms, Drug Resistance and Therapeutic Implications. *J. Clin. Med.* **2023**, *12*, 7442. https://doi.org/10.3390/jcm12237442.
16. Parakh, S.; Nicolazzo, J.; Scott, A.M.; Gan, H.K. Antibody Drug Conjugates in Glioblastoma–Is There a Future for Them? *Front. Oncol.* **2021**, *11*, 718590. Available online: https://www.frontiersin.org/articles/10.3389/fonc.2021.718590 (accessed on 3 December 2021).
17. Hansson, J.; Martenhed, G.; Egyházi, S.; Tani, E.; Platz, A. Analysis of O6-methylguanine-DNA methyltransferase mRNA in fine needle biopsies from human melanoma metastases by reverse transcription and polymerase chain reaction. *Eur. J. Cancer* **1996**, *32*, 2319–2326. https://doi.org/10.1016/S0959-8049(96)00304-8.
18. Fuster-Garcia, E.; Lorente Estellés, D.; del Mar Álvarez-Torres, M.; Juan-Albarracín, J.; Chelebian, E.; Rovira, A.; Acosta, C.A.; Pineda, J.; Oleaga, L.; Mollá-Olmos, E.; et al. MGMT methylation may benefit overall survival in patients with moderately vascularized glioblastomas. *Eur. Radiol.* **2021**, *31*, 1738–1747. https://doi.org/10.1007/s00330-020-07297-4.
19. Perazzoli, G.; Prados, J.; Ortiz, R.; Caba, O.; Cabeza, L.; Berdasco, M.; Gónzalez, B.; Melguizo, C. Temozolomide Resistance in Glioblastoma Cell Lines: Implication of MGMT, MMR, P-Glycoprotein and CD133 Expression. *PLoS ONE* **2015**, *10*, e0140131. https://doi.org/10.1371/journal.pone.0140131.
20. Hunter, C.; Smith, R.; Cahill, D.P.; Stephens, P.; Stevens, C.; Teague, J.; Greenman, C.; Edkins, S.; Bignell, G.; Davies, H.; et al. A Hypermutation Phenotype and Somatic MSH6 Mutations in Recurrent Human Malignant Gliomas after Alkylator Chemotherapy. *Cancer Res.* **2006**, *66*, 3987–3991. https://doi.org/10.1158/0008-5472.CAN-06-0127.

21. Li, J.; Koczor, C.A.; Saville, K.M.; Hayat, F.; Beiser, A.; McClellan, S.; Migaud, M.E.; Sobol, R.W. Overcoming Temozolomide Resistance in Glioblastoma via Enhanced NAD+ Bioavailability and Inhibition of Poly-ADP-Ribose Glycohydrolase. *Cancers* **2022**, *14*, 3572. https://doi.org/10.3390/cancers14153572.
22. Stark, A.M.; Doukas, A.; Hugo, H.-H.; Hedderich, J.; Hattermann, K.; Maximilian Mehdorn, H.; Held-Feindt, J. Expression of DNA mismatch repair proteins MLH1, MSH2, and MSH6 in recurrent glioblastoma. *Neurol. Res.* **2015**, *37*, 95–105. https://doi.org/10.1179/1743132814Y.0000000409.
23. Kinsella, T.J. Coordination of DNA Mismatch Repair and Base Excision Repair Processing of Chemotherapy and Radiation Damage for Targeting Resistant Cancers. *Clin. Cancer Res.* **2009**, *15*, 1853–1859. https://doi.org/10.1158/1078-0432.CCR-08-1307.
24. Wang, J.Y.J.; Edelmann, W. Mismatch repair proteins as sensors of alkylation DNA damage. *Cancer Cell* **2006**, *9*, 417–418. https://doi.org/10.1016/j.ccr.2006.05.013.
25. Yoshimoto, K.; Mizoguchi, M.; Hata, N.; Murata, H.; Hatae, R.; Amano, T.; Nakamizo, A.; Sasaki, T. Complex DNA repair pathways as possible therapeutic targets to overcome temozolomide resistance in glioblastoma. *Front. Oncol.* **2012**, *2*, 186. Available online: https://www.frontiersin.org/articles/10.3389/fonc.2012.00186 (accessed on).
26. Zampieri, L.X.; Sboarina, M.; Cacace, A.; Grasso, D.; Thabault, L.; Hamelin, L.; Vazeille, T.; Dumon, E.; Rossignol, R.; Frédérick, R.; et al. Olaparib Is a Mitochondrial Complex I Inhibitor That Kills Temozolomide-Resistant Human Glioblastoma Cells. *Int. J. Mol. Sci.* **2021**, *22*, 11938. https://doi.org/10.3390/ijms222111938.
27. Trivedi, R.N.; Almeida, K.H.; Fornsaglio, J.L.; Schamus, S.; Sobol, R.W. The Role of Base Excision Repair in the Sensitivity and Resistance to Temozolomide-Mediated Cell Death. *Cancer Res.* **2005**, *65*, 6394–6400. https://doi.org/10.1158/0008-5472.CAN-05-0715.
28. Strik, H.M.; Schmidt, K.; Lingor, P.; Tönges, L.; Kugler, W.; Nitsche, M.; Rabinovich, G.A.; Bähr, M. Galectin-1 expression in human glioma cells: Modulation by ionizing radiation and effects on tumor cell proliferation and migration. *Oncol. Rep.* **2007**, *18*, 483–488. https://doi.org/10.3892/or.18.2.483.
29. Sachdeva, R.; Wu, M.; Smiljanic, S.; Kaskun, O.; Ghannad-Zadeh, K.; Celebre, A.; Isaev, K.; Morrissy, A.S.; Guan, J.; Tong, J.; et al. ID1 Is Critical for Tumorigenesis and Regulates Chemoresistance in Glioblastoma. *Cancer Res.* **2019**, *79*, 4057–4071. https://doi.org/10.1158/0008-5472.CAN-18-1357.
30. Ranjan, A.; Kaushik, I.; Srivastava, S.K. Pimozide Suppresses the Growth of Brain Tumors by Targeting STAT3-Mediated Autophagy. *Cells* **2020**, *9*, 2141. https://doi.org/10.3390/cells9092141.
31. Barthel, L.; Hadamitzky, M.; Dammann, P.; Schedlowski, M.; Sure, U.; Thakur, B.K.; Hetze, S. Glioma: Molecular signature and crossroads with tumor microenvironment. *Cancer Metast. Rev.* **2022**, *41*, 53–75. https://doi.org/10.1007/s10555-021-09997-9.
32. Hambardzumyan, D.; Gutmann, D.H.; Kettenmann, H. The role of microglia and macrophages in glioma maintenance and progression. *Nat. Neurosci.* **2016**, *19*, 20–27. https://doi.org/10.1038/nn.4185.
33. Hung, H.-C.; Liu, C.-C.; Chuang, J.-Y.; Su, C.-L.; Gean, P.-W. Inhibition of Sonic Hedgehog Signaling Suppresses Glioma Stem-Like Cells Likely Through Inducing Autophagic Cell Death. *Front. Oncol.* **2020**, *10*, 1233. Available online: https://www.frontiersin.org/articles/10.3389/fonc.2020.01233 (accessed on).
34. Saito, N.; Aoki, K.; Hirai, N.; Fujita, S.; Iwama, J.; Hiramoto, Y.; Ishii, M.; Sato, K.; Nakayama, H.; Harashina, J.; et al. Effect of Notch expression in glioma stem cells on therapeutic response to chemo-radiotherapy in recurrent glioblastoma. *Brain Tumor Pathol.* **2015**, *32*, 176–183. https://doi.org/10.1007/s10014-015-0215-7.
35. Yao, X.; Ping, Y.; Liu, Y.; Chen, K.; Yoshimura, T.; Liu, M.; Gong, W.; Chen, C.; Niu, Q.; Guo, D.; et al. Vascular Endothelial Growth Factor Receptor 2 (VEGFR-2) Plays a Key Role in Vasculogenic Mimicry Formation, Neovascularization and Tumor Initiation by Glioma Stem-like Cells. *PLoS ONE* **2013**, *8*, e57188. https://doi.org/10.1371/journal.pone.0057188.
36. Ameratunga, M.; Pavlakis, N.; Wheeler, H.; Grant, R.; Simes, J.; Khasraw, M. Anti-angiogenic therapy for high-grade glioma. *Cochrane Database Syst. Rev.* **2018**, *018*, CD008218. https://doi.org/10.1002/14651858.CD008218.pub4.
37. Wang, G.; Zhong, K.; Wang, Z.; Zhang, Z.; Tang, X.; Tong, A.; Zhou, L. Tumor-associated microglia and macrophages in glioblastoma: From basic insights to therapeutic opportunities. *Front. Immunol.* **2022**, *13*, 964898. Available online: https://www.frontiersin.org/articles/10.3389/fimmu.2022.964898 (accessed on).
38. Geribaldi-Doldán, N.; Fernández-Ponce, C.; Quiroz, R.N.; Sánchez-Gomar, I.; Escorcia, L.G.; Velásquez, E.P.; Quiroz, E.N. The Role of Microglia in Glioblastoma. *Front. Oncol.* **2021**, *10*, 603495. Available online: https://www.frontiersin.org/articles/10.3389/fonc.2020.603495 (accessed on).
39. Bowman, R.L.; Joyce, J.A. Therapeutic targeting of tumor-associated macrophages and microglia in glioblastoma. *Immunotherapy* **2014**, *6*, 663–666. https://doi.org/10.2217/imt.14.48.
40. Virga, J.; Szivos, L.; Hortobágyi, T.; Chalsaraei, M.K.; Zahuczky, G.; Steiner, L.; Tóth, J.; Reményi-Puskár, J.; Bognár, L.; Klekner, A. Extracellular matrix differences in glioblastoma patients with different prognoses. *Oncol. Lett.* **2019**, *17*, 797–806. https://doi.org/10.3892/ol.2018.9649.
41. Kim, Y.; Kang, H.; Powathil, G.; Kim, H.; Trucu, D.; Lee, W.; Lawler, S.; Chaplain, M. Role of extracellular matrix and microenvironment in regulation of tumor growth and LAR-mediated invasion in glioblastoma. *PLoS ONE* **2018**, *13*, e0204865. https://doi.org/10.1371/journal.pone.0204865.
42. Chistiakov, D.A.; Chekhonin, V.P. Extracellular vesicles shed by glioma cells: Pathogenic role and clinical value. *Tumor Biol.* **2014**, *35*, 8425–8438. https://doi.org/10.1007/s13277-014-2262-9.
43. Guo, X.; Sui, R.; Piao, H. Tumor-derived small extracellular vesicles: Potential roles and mechanism in glioma. *J. Nanobiotechnol.* **2022**, *20*, 383. https://doi.org/10.1186/s12951-022-01584-6.

44. Nandhu, M.S.; Behera, P.; Bhaskaran, V.; Longo, S.L.; Barrera-Arenas, L.M.; Sengupta, S.; Rodriguez-Gil, D.J.; Chiocca, E.A.; Viapiano, M.S. Development of a Function-Blocking Antibody Against Fibulin-3 as a Targeted Reagent for Glioblastoma. *Clin. Cancer Res.* **2018**, *24*, 821–833. https://doi.org/10.1158/1078-0432.CCR-17-1628.
45. Spaeth, N.; Wyss, M.T.; Pahnke, J.; Biollaz, G.; Trachsel, E.; Drandarov, K.; Treyer, V.; Weber, B.; Neri, D.; Buck, A. Radioimmunotherapy targeting the extra domain B of fibronectin in C6 rat gliomas: A preliminary study about the therapeutic efficacy of iodine-131-labeled SIP(L19). *Nucl. Med. Biol.* **2006**, *33*, 661–666. https://doi.org/10.1016/j.nucmedbio.2006.05.001.
46. Brack, S.S.; Silacci, M.; Birchler, M.; Neri, D. Tumor-Targeting Properties of Novel Antibodies Specific to the Large Isoform of Tenascin-C. *Clin. Cancer Res.* **2006**, *12*, 3200–3208. https://doi.org/10.1158/1078-0432.CCR-05-2804.
47. Lingasamy, P.; Tobi, A.; Haugas, M.; Hunt, H.; Paiste, P.; Asser, T.; Rätsep, T.; Kotamraju, V.R.; Bjerkvig, R.; Teesalu, T. Bi-specific tenascin-C and fibronectin targeted peptide for solid tumor delivery. *Biomaterials* **2019**, *219*, 119373. https://doi.org/10.1016/j.biomaterials.2019.119373.
48. Zukiel, R.; Nowak, S.; Wyszko, E.; Rolle, K.; Gawronska, I.; Barciszewska, M.Z.; Barciszewski, J. Suppression of human brain tumor with interference RNA specific for tenascin-C. *Cancer Biol. Ther.* **2006**, *5*, 1002–1007. https://doi.org/10.4161/cbt.5.8.2886.
49. Sette, P.; Amankulor, N.; Li, A.; Marzulli, M.; Leronni, D.; Zhang, M.; Goins, W.F.; Kaur, B.; Bolyard, C.; Cripe, T.P.; et al. GBM-Targeted oHSV Armed with Matrix Metalloproteinase 9 Enhances Anti-tumor Activity and Animal Survival. *Mol. Ther.-Oncolyt.* **2019**, *15*, 214–222. https://doi.org/10.1016/j.omto.2019.10.005.
50. Jiang, X.; Zhou, T.; Wang, Z.; Qi, B.; Xia, H. HSP47 Promotes Glioblastoma Stemlike Cell Survival by Modulating Tumor Microenvironment Extracellular Matrix through TGF-β Pathway. *ACS Chem. Neurosci.* **2017**, *8*, 128–134. https://doi.org/10.1021/acschemneuro.6b00253.
51. Jaime-Ramirez, A.C.; Dmitrieva, N.; Yoo, J.Y.; Banasavadi-Siddegowda, Y.; Zhang, J.; Relation, T.; Bolyard, C.; Wojton, J.; Kaur, B. Humanized chondroitinase ABC sensitizes glioblastoma cells to temozolomide. *J. Gene Med.* **2017**, *19*, e2942. https://doi.org/10.1002/jgm.2942.
52. Rajesh, Y.; Biswas, A.; Kumar, U.; Banerjee, I.; Das, S.; Maji, S.; Das, S.K.; Emdad, L.; Cavenee, W.K.; Mandal, M.; et al. Lumefantrine, an antimalarial drug, reverses radiation and temozolomide resistance in glioblastoma. *Proc. Natl. Acad. Sci. USA* **2020**, *117*, 12324–12331. https://doi.org/10.1073/pnas.1921531117.
53. Gwak, H.-S.; Park, M.-J.; Park, I.-C.; Woo, S.H.; Jin, H.-O.; Rhee, C.H.; Jung, H.-W. Tetraarsenic oxide–induced inhibition of malignant glioma cell invasion in vitro via a decrease in matrix metalloproteinase secretion and protein kinase B phosphorylation: Laboratory investigation. *J. Neurosurg. JNS* **2014**, *121*, 1483–1491. https://doi.org/10.3171/2014.8.JNS131991.
54. Alafate, W.; Wang, M.; Zuo, J.; Wu, W.; Sun, L.; Liu, C.; Xie, W.; Wang, J. Targeting Aurora kinase B attenuates chemoresistance in glioblastoma via a synergistic manner with temozolomide. *Pathol.-Res. Pract.* **2019**, *215*, 152617. https://doi.org/10.1016/j.prp.2019.152617.
55. Yun, E.-J.; Kim, S.; Hsieh, J.-T.; Baek, S.T. Wnt/β-catenin signaling pathway induces autophagy-mediated temozolomide-resistance in human glioblastoma. *Cell Death Dis.* **2020**, *11*, 771. https://doi.org/10.1038/s41419-020-02988-8.
56. Ou, A.; Ott, M.; Fang, D.; Heimberger, A.B. The Role and Therapeutic Targeting of JAK/STAT Signaling in Glioblastoma. *Cancers* **2021**, *13*, 437. https://doi.org/10.3390/cancers13030437.
57. Monteiro, A.R.; Hill, R.; Pilkington, G.J.; Madureira, P.A. The Role of Hypoxia in Glioblastoma Invasion. *Cells* **2017**, *6*, 45. https://doi.org/10.3390/cells6040045.
58. Park, J.H.; Lee, H.K. Current Understanding of Hypoxia in Glioblastoma Multiforme and Its Response to Immunotherapy. *Cancers* **2022**, *14*, 1176. https://doi.org/10.3390/cancers14051176.
59. Rao, J.U.; Coman, D.; Walsh, J.J.; Ali, M.M.; Huang, Y.; Hyder, F. Temozolomide arrests glioma growth and normalizes intratumoral extracellular pH. *Sci. Rep.* **2017**, *7*, 7865. https://doi.org/10.1038/s41598-017-07609-7.
60. Ranjit, M.; Hirano, M.; Aoki, K.; Okuno, Y.; Ohka, F.; Yamamichi, A.; Kato, A.; Maeda, S.; Motomura, K.; Matsuo, K.; et al. Aberrant Active cis-Regulatory Elements Associated with Downregulation of RET Finger Protein Overcome Chemoresistance in Glioblastoma. *Cell Rep.* **2019**, *26*, 2274–2281.e5. https://doi.org/10.1016/j.celrep.2019.01.109.
61. Hirano, M.; Ranjit, M.; Yamamichi, A.; Aoki, K.; Ohka, F.; Kato, T.; Enomoto, A.; Takahashi, M.; Wakabayashi, T.; Natsume, A. GENE-49. Aberrant super-enhancers associated with downregulation of ret finger protein overcomes chemoresistance in glioblastoma. *Neuro-Oncology* **2017**, *19* (Suppl. 6), vi103. https://doi.org/10.1093/neuonc/nox168.421.
62. Li, H.; Chen, L.; Li, J.; Zhou, Q.; Huang, A.; Liu, W.; Wang, K.; Gao, L.; Qi, S.; Lu, Y. miR-519a enhances chemosensitivity and promotes autophagy in glioblastoma by targeting STAT3/Bcl2 signaling pathway. *J. Hematol. Oncol.* **2018**, *11*, 70. https://doi.org/10.1186/s13045-018-0618-0.
63. Cardoso, A.M.; Morais, C.M.; Sousa, M.; Rebelo, O.; Tão, H.; Barbosa, M.; Pedroso de Lima, M.C.; Jurado, A.S. MiR-200c-based metabolic modulation in glioblastoma cells as a strategy to overcome tumor chemoresistance. *Hum. Mol. Genet.* **2021**, *30*, 2315–2331. https://doi.org/10.1093/hmg/ddab193.
64. Li, N.; Maly, D.J.; Chanthery, Y.H.; Sirkis, D.W.; Nakamura, J.L.; Berger, M.S.; James, C.D.; Shokat, K.M.; Weiss, W.A.; Persson, A.I. Radiotherapy Followed by Aurora Kinase Inhibition Targets Tumor-Propagating Cells in Human Glioblastoma. *Mol. Cancer Ther.* **2015**, *14*, 419–428. https://doi.org/10.1158/1535-7163.MCT-14-0526.
65. Zhan, X.; Qiu, R.; He, Y.; Zhao, Z.; Huang, M.; Liu, Q.; Zhi, F.; Long, W. The Aurora Kinase Inhibitor TAK901 Inhibits Glioblastoma Growth by Blocking SREBP1-Mediated Lipid Metabolism. *Cancers* **2022**, *14*, 5805. https://doi.org/10.3390/cancers14235805.
66. Shireman, J.M.; Atashi, F.; Lee, G.; Ali, E.S.; Saathoff, M.R.; Park, C.H.; Savchuk, S.; Baisiwala, S.; Miska, J.; Lesniak, M.S.; et al. De novo purine biosynthesis is a major driver of chemoresistance in glioblastoma. *Brain* **2021**, *144*, 1230–1246. https://doi.org/10.1093/brain/awab020.

67. Zhou, W.; Yao, Y.; Scott, A.J.; Wilder-Romans, K.; Dresser, J.J.; Werner, C.K.; Sun, H.; Pratt, D.; Sajjakulnukit, P.; Zhao, S.G.; et al. Purine metabolism regulates DNA repair and therapy resistance in glioblastoma. *Nat. Commun.* **2020**, *11*, 3811. https://doi.org/10.1038/s41467-020-17512-x.
68. Friedmann-Morvinski, D.; Narasimamurthy, R.; Xia, Y.; Myskiw, C.; Soda, Y.; Verma, I.M. Targeting NF-κB in glioblastoma: A therapeutic approach. *Sci. Adv.* **2016**, *2*, e1501292. https://doi.org/10.1126/sciadv.1501292.
69. Yu, Z.; Chen, Y.; Wang, S.; Li, P.; Zhou, G.; Yuan, Y. Inhibition of NF-κB results in anti-glioma activity and reduces temozolomide-induced chemoresistance by down-regulating MGMT gene expression. *Cancer Lett.* **2018**, *428*, 77–89. https://doi.org/10.1016/j.canlet.2018.04.033.
70. Avci, N.G.; Ebrahimzadeh-Pustchi, S.; Akay, Y.M.; Esquenazi, Y.; Tandon, N.; Zhu, J.-J.; Akay, M. NF-κB inhibitor with Temozolomide results in significant apoptosis in glioblastoma via the NF-κB(p65) and actin cytoskeleton regulatory pathways. *Sci. Rep.* **2020**, *10*, 13352. https://doi.org/10.1038/s41598-020-70392-5.
71. Cheng, Y.Y.; Yang, X.; Gao, X.; Song, S.X.; Yang, M.F.; Xie, F.M. LGR6 promotes glioblastoma malignancy and chemoresistance by activating the Akt signaling pathway. *Exp. Ther. Med.* **2021**, *22*, 1364. https://doi.org/10.3892/etm.2021.10798.
72. Zhang, J.; Chen, G.; Gao, Y.; Liang, H. HOTAIR/miR-125 axis-mediated Hexokinase 2 expression promotes chemoresistance in human glioblastoma. *J. Cell. Mol. Med.* **2020**, *24*, 5707–5717. https://doi.org/10.1111/jcmm.15233.
73. Wei, Y.; Lu, C.; Zhou, P.; Zhao, L.; Lyu, X.; Yin, J.; Shi, Z.; You, Y. EIF4A3-induced circular RNA ASAP1 promotes tumorigenesis and temozolomide resistance of glioblastoma via NRAS/MEK1/ERK1–2 signaling. *Neuro-Oncology* **2021**, *23*, 611–624. https://doi.org/10.1093/neuonc/noaa214.
74. Wang, X.; Liu, T.; Bai, Y.; Liao, H.; Qiu, S.; Chang, Z.; Liu, Y.; Yan, X.; Guo, H. Polymerase I and Transcript Release Factor Acts As an Essential Modulator of Glioblastoma Chemoresistance. *PLoS ONE* **2014**, *9*, e93439. https://doi.org/10.1371/journal.pone.0093439.
75. Yang, E.; Wang, L.; Jin, W.; Liu, X.; Wang, Q.; Wu, Y.; Tan, Y.; Wang, Y.; Cui, X.; Zhao, J.; et al. PTRF/Cavin-1 enhances chemo-resistance and promotes temozolomide efflux through extracellular vesicles in glioblastoma. *Theranostics* **2022**, *12*, 4330–4347. https://doi.org/10.7150/thno.71763.
76. Yang, L.; Zhang, H. Expression of Cytosolic Phospholipase A2 Alpha in Glioblastoma Is Associated With Resistance to Chemotherapy. *Am. J. Med. Sci.* **2018**, *356*, 391–398. https://doi.org/10.1016/j.amjms.2018.06.019.
77. Mahinfar, P.; Baradaran, B.; Davoudian, S.; Vahidian, F.; Cho, W.C.; Mansoori, B. Long Non-Coding RNAs in Multidrug Resistance of Glioblastoma. *Genes* **2021**, *12*, 455. https://doi.org/10.3390/genes12030455.
78. Li, Z.; Meng, X.; Wu, P.; Zha, C.; Han, B.; Li, L.; Sun, N.; Qi, T.; Qin, J.; Zhang, Y.; et al. Glioblastoma Cell–Derived lncRNA-Containing Exosomes Induce Microglia to Produce Complement C5, Promoting Chemotherapy Resistance. *Cancer Immunol. Res.* **2021**, *9*, 1383–1399. https://doi.org/10.1158/2326-6066.CIR-21-0258.
79. Azuaje, F.; Tiemann, K.; Niclou, S.P. Therapeutic control and resistance of the EGFR-driven signaling network in glioblastoma. *Cell Commun. Signal.* **2015**, *13*, 23. https://doi.org/10.1186/s12964-015-0098-6.
80. Westphal, M.; Maire, C.L.; Lamszus, K. EGFR as a Target for Glioblastoma Treatment: An Unfulfilled Promise. *CNS Drugs* **2017**, *31*, 723–735. https://doi.org/10.1007/s40263-017-0456-6.
81. E. Taylor, T.; B. Furnari, F.; K. Cavenee, W. Targeting EGFR for Treatment of Glioblastoma: Molecular Basis to Overcome Resistance. *Curr. Cancer Drug Targets* **2012**, *12*, 197–209. https://doi.org/10.2174/156800912799277557.
82. Wen, P.Y.; Lee, E.Q.; Reardon, D.A.; Ligon, K.L.; Alfred Yung, W.K. Current clinical development of PI3K pathway inhibitors in glioblastoma. *Neuro-Oncology* **2012**, *14*, 819–829. https://doi.org/10.1093/neuonc/nos117.
83. Hainsworth, J.D.; Becker, K.P.; Mekhail, T.; Chowdhary, S.A.; Eakle, J.F.; Wright, D.; Langdon, R.M.; Yost, K.J.; Padula, G.D.A.; West-Osterfield, K.; et al. Phase I/II study of bevacizumab with BKM120, an oral PI3K inhibitor, in patients with refractory solid tumors (phase I) and relapsed/refractory glioblastoma (phase II). *J. Neuro-Oncol.* **2019**, *144*, 303–311. https://doi.org/10.1007/s11060-019-03227-7.
84. Kaminska, B.; Cyranowski, S. Recent Advances in Understanding Mechanisms of TGF Beta Signaling and Its Role in Glioma Pathogenesis. In *Glioma Signaling*; Barańska, J., Ed.; Springer International Publishing: Cham, Switzerland, 2020; pp. 179–201, ISBN 978-3-030-30651-9.
85. Towner, R.A.; Smith, N.; Saunders, D.; Brown, C.A.; Cai, X.; Ziegler, J.; Mallory, S.; Dozmorov, M.G.; Coutinho De Souza, P.; Wiley, G.; et al. OKN-007 Increases temozolomide (TMZ) Sensitivity and Suppresses TMZ-Resistant Glioblastoma (GBM) Tumor Growth. *Transl. Oncol.* **2019**, *12*, 320–335. https://doi.org/10.1016/j.tranon.2018.10.002.
86. Bogdahn, U.; Hau, P.; Stockhammer, G.; Venkataramana, N.K.; Mahapatra, A.K.; Suri, A.; Balasubramaniam, A.; Nair, S.; Oliushine, V.; Parfenov, V.; et al. Targeted therapy for high-grade glioma with the TGF-β2 inhibitor trabedersen: Results of a randomized and controlled phase IIb study. *Neuro-Oncology* **2011**, *13*, 132–142. https://doi.org/10.1093/neuonc/noq142.
87. Tashima, T. Brain Cancer Chemotherapy through a Delivery System across the Blood-Brain Barrier into the Brain Based on Receptor-Mediated Transcytosis Using Monoclonal Antibody Conjugates. *Biomedicines* **2022**, *10*, 1597. https://doi.org/10.3390/biomedicines10071597.
88. Riva, P.; Franceschi, G.; Frattarelli, M.; Riva, N.; Guiducci, G.; Cremonini, A.M.; Giuliani, G.; Casi, M.; Gentile, R.; Jekunen, A.A.; et al. 131I Radioconjugated Antibodies for the Locoregional Radioimmunotherapy of High-grade Malignant Glioma: Phase I and II Study. *Acta Oncol.* **1999**, *38*, 351–359. https://doi.org/10.1080/028418699431438.
89. Reardon, D.A.; Zalutsky, M.R.; Akabani, G.; Coleman, R.E.; Friedman, A.H.; Herndon, J.E., II; McLendon, R.E.; Pegram, C.N.; Quinn, J.A.; Rich, J.N.; et al. A pilot study: 131I-Antitenascin monoclonal antibody 81c6 to deliver a 44-Gy resection cavity boost. *Neuro-Oncology* **2008**, *10*, 182–189. https://doi.org/10.1215/15228517-2007-053.

90. Li, L.; Quang, T.S.; Gracely, E.J.; Kim, J.H.; Emrich, J.G.; Yaeger, T.E.; Jenrette, J.M.; Cohen, S.C.; Black, P.; Brady, L.W. A Phase II study of anti–epidermal growth factor receptor radioimmunotherapy in the treatment of glioblastoma multiforme: Clinical article. *J. Neurosurg. JNS* **2010**, *113*, 192–198. https://doi.org/10.3171/2010.2.JNS091211.
91. Weaver, M.; Laske, D.W. Transferrin Receptor Ligand-Targeted Toxin Conjugate (Tf-CRM107) for Therapy of Malignant Gliomas. *J. Neuro-Oncol.* **2003**, *65*, 3–14. https://doi.org/10.1023/A:1026246500788.
92. Sampson, J.H.; Akabani, G.; Archer, G.E.; Berger, M.S.; Coleman, R.E.; Friedman, A.H.; Friedman, H.S.; Greer, K.; Herndon, J.E., II; Kunwar, S.; et al. Intracerebral infusion of an EGFR-targeted toxin in recurrent malignant brain tumors. *Neuro-Oncology* **2008**, *10*, 320–329. https://doi.org/10.1215/15228517-2008-012.
93. Kunwar, S.; Chang, S.; Westphal, M.; Vogelbaum, M.; Sampson, J.; Barnett, G.; Shaffrey, M.; Ram, Z.; Piepmeier, J.; Prados, M.; et al. Phase III randomized trial of CED of IL13-PE38QQR vs Gliadel wafers for recurrent glioblastoma†. *Neuro-Oncology* **2010**, *12*, 871–881. https://doi.org/10.1093/neuonc/nop054.
94. Weber, F.; Asher, A.; Bucholz, R.; Berger, M.; Prados, M.; Chang, S.; Bruce, J.; Hall, W.; Rainov, N.G.; Westphal, M.; et al. Safety, tolerability, and tumor response of IL4-Pseudomonas exotoxin (NBI-3001) in patients with recurrent malignant glioma. *J. Neuro-Oncol.* **2003**, *64*, 125–137. https://doi.org/10.1007/BF02700027.
95. Reardon, D.A.; Lassman, A.B.; van den Bent, M.; Kumthekar, P.; Merrell, R.; Scott, A.M.; Fichtel, L.; Sulman, E.P.; Gomez, E.; Fischer, J.; et al. Efficacy and safety results of ABT-414 in combination with radiation and temozolomide in newly diagnosed glioblastoma. *Neuro-Oncology* **2017**, *19*, 965–975. https://doi.org/10.1093/neuonc/now257.
96. Gan, H.K.; Fichtel, L.; Lassman, A.B.; Merrell, R.; van den Bent, M.; Kumthekar, P.; Scott, A.M.; Pedersen, M.; Gomez, E.; Fischer, J.; et al. ET-19A phase 1 study evaluating ABT-414 with temozolomide (TMZ) or concurrent radiotherapy (RT) and TMZ in glioblastoma (GBM). *Neuro-Oncology* **2014**, *16* (Suppl. 5), v83. https://doi.org/10.1093/neuonc/nou255.19.
97. Rosenthal, M.; Curry, R.; Reardon, D.A.; Rasmussen, E.; Upreti, V.V.; Damore, M.A.; Henary, H.A.; Hill, J.S.; Cloughesy, T. Safety, tolerability, and pharmacokinetics of anti-EGFRvIII antibody–drug conjugate AMG 595 in patients with recurrent malignant glioma expressing EGFRvIII. *Cancer Chemother. Pharmacol.* **2019**, *84*, 327–336. https://doi.org/10.1007/s00280-019-03879-2.
98. Wang, Y.; Long, P.; Wang, Y.; Ma, W. NTRK Fusions and TRK Inhibitors: Potential Targeted Therapies for Adult Glioblastoma. *Front. Oncol.* **2020**, *10*, 593578. Available online: https://www.frontiersin.org/articles/10.3389/fonc.2020.593578 (accessed on).
99. Grogan, P.T.; Deming, D.A.; Helgager, J.; Ruszkiewicz, T.; Baskaya, M.K.; Howard, S.P.; Robins, H.I. Entrectinib demonstrates prolonged efficacy in an adult case of radiation-refractory NTRK fusion glioblastoma. *Neuro-Oncol. Adv.* **2022**, *4*, vdac046. https://doi.org/10.1093/noajnl/vdac046.
100. Rayi, A.; Alnahhas, I.; Ong, S.; Giglio, P.; Puduvalli, V.K. Targeted Therapy for BRAF Mutant Brain Tumors. *Curr. Treat. Options Oncol.* **2021**, *22*, 105. https://doi.org/10.1007/s11864-021-00901-9.
101. Burger, M.C.; Ronellenfitsch, M.W.; Lorenz, N.I.; Wagner, M.; Voss, M.; Capper, D.; Tzaridis, T.; Herrlinger, U.; Steinbach, J.P.; Stoffels, G.; et al. Dabrafenib in patients with recurrent, BRAF V600E mutated malignant glioma and leptomeningeal disease. *Oncol. Rep.* **2017**, *38*, 3291–3296. https://doi.org/10.3892/or.2017.6013.
102. Wen, P.Y.; Stein, A.; van den Bent, M.; De Greve, J.; Wick, A.; de Vos, F.Y.F.L.; von Bubnoff, N.; van Linde, M.E.; Lai, A.; Prager, G.W.; et al. Dabrafenib plus trametinib in patients with BRAFV600E-mutant low-grade and high-grade glioma (ROAR): A multicentre, open-label, single-arm, phase 2, basket trial. *Lancet Oncol.* **2022**, *23*, 53–64. https://doi.org/10.1016/S1470-2045(21)00578-7.
103. Kaley, T.; Touat, M.; Subbiah, V.; Hollebecque, A.; Rodon, J.; Lockhart, A.C.; Keedy, V.; Bielle, F.; Hofheinz, R.-D.; Joly, F.; et al. BRAF Inhibition in BRAFV600-Mutant Gliomas: Results From the VE-BASKET Study. *J. Clin. Oncol.* **2018**, *36*, 3477–3484. https://doi.org/10.1200/JCO.2018.78.9990.
104. Di Filippo, L.D.; Lobato Duarte, J.; Hofstätter Azambuja, J.; Isler Mancuso, R.; Tavares Luiz, M.; Hugo Sousa Araújo, V.; Delbone Figueiredo, I.; Barretto-de-Souza, L.; Miguel Sábio, R.; Sasso-Cerri, E.; et al. Glioblastoma multiforme targeted delivery of docetaxel using bevacizumab-modified nanostructured lipid carriers impair in vitro cell growth and in vivo tumor progression. *Int. J. Pharm.* **2022**, *618*, 121682. https://doi.org/10.1016/j.ijpharm.2022.121682.
105. Rong, L.; Li, N.; Zhang, Z. Emerging therapies for glioblastoma: Current state and future directions. *J. Exp. Clin. Cancer Res.* **2022**, *41*, 142. https://doi.org/10.1186/s13046-022-02349-7.
106. O◆Rourke, D.M.; Nasrallah, M.P.; Desai, A.; Melenhorst, J.J.; Mansfield, K.; Morrissette, J.J.D.; Martinez-Lage, M.; Brem, S.; Maloney, E.; Shen, A.; et al. A single dose of peripherally infused EGFRvIII-directed CAR T cells mediates antigen loss and induces adaptive resistance in patients with recurrent glioblastoma. *Sci. Transl. Med.* **2017**, *9*, eaaa0984. https://doi.org/10.1126/scitranslmed.aaa0984.
107. Wei, J.; Wang, Z.; Wang, W.; Liu, X.; Wan, J.; Yuan, Y.; Li, X.; Ma, L.; Liu, X. Oxidative Stress Activated by Sorafenib Alters the Temozolomide Sensitivity of Human Glioma Cells Through Autophagy and JAK2/STAT3-AIF Axis. *Front. Cell Dev. Biol.* **2021**, *9*, 660005. Available online: https://www.frontiersin.org/articles/10.3389/fcell.2021.660005 (accessed on).
108. Hottinger, A.F.; Aissa, A.B.; Espeli, V.; Squiban, D.; Dunkel, N.; Vargas, M.I.; Hundsberger, T.; Mach, N.; Schaller, K.; Weber, D.C.; et al. Phase I study of sorafenib combined with radiation therapy and temozolomide as first-line treatment of high-grade glioma. *Br. J. Cancer* **2014**, *110*, 2655–2661. https://doi.org/10.1038/bjc.2014.209.
109. Chen, H.; Kuhn, J.; Lamborn, K.R.; Abrey, L.E.; DeAngelis, L.M.; Lieberman, F.; Robins, H.I.; Chang, S.M.; Yung, W.K.A.; Drappatz, J.; et al. Phase I/II study of sorafenib in combination with erlotinib for recurrent glioblastoma as part of a 3-arm sequential accrual clinical trial: NABTC 05-02. *Neuro-Oncol. Adv.* **2020**, *2*, vdaa124. https://doi.org/10.1093/noajnl/vdaa124.

110. de la Fuente, M.I.; Colman, H.; Rosenthal, M.; Van Tine, B.A.; Levacic, D.; Walbert, T.; Gan, H.K.; Vieito, M.; Milhem, M.M.; Lipford, K.; et al. Olutasidenib (FT-2102) in patients with relapsed or refractory IDH1-mutant glioma: A multicenter, open-label, phase Ib/II trial. *Neuro-Oncology* **2023**, *25*, 146–156. https://doi.org/10.1093/neuonc/noac139.
111. Mellinghoff, I.K.; Penas-Prado, M.; Peters, K.B.; Burris, H.A., III; Maher, E.A.; Janku, F.; Cote, G.M.; de la Fuente, M.I.; Clarke, J.L.; Ellingson, B.M.; et al. Vorasidenib, a Dual Inhibitor of Mutant IDH1/2, in Recurrent or Progressive Glioma; Results of a First-in-Human Phase I Trial. *Clin. Cancer Res.* **2021**, *27*, 4491–4499. https://doi.org/10.1158/1078-0432.CCR-21-0611.
112. Mellinghoff, I.K.; Lu, M.; Wen, P.Y.; Taylor, J.W.; Maher, E.A.; Arrillaga-Romany, I.; Peters, K.B.; Ellingson, B.M.; Rosenblum, M.K.; Chun, S.; et al. Vorasidenib and ivosidenib in IDH1-mutant low-grade glioma: A randomized, perioperative phase 1 trial. *Nat. Med.* **2023**, *29*, 615–622. https://doi.org/10.1038/s41591-022-02141-2.
113. Punganuru, S.R.; Arutla, V.; Zhao, W.; Rajaei, M.; Deokar, H.; Zhang, R.; Buolamwini, J.K.; Srivenugopal, K.S.; Wang, W. Targeted Brain Tumor Therapy by Inhibiting the MDM2 Oncogene: In Vitro and In Vivo Antitumor Activity and Mechanism of Action. *Cells* **2020**, *9*, 1592. https://doi.org/10.3390/cells9071592.
114. Lombardi, G.; De Salvo, G.L.; Brandes, A.A.; Eoli, M.; Rudà, R.; Faedi, M.; Lolli, I.; Pace, A.; Daniele, B.; Pasqualetti, F.; et al. Regorafenib compared with lomustine in patients with relapsed glioblastoma (REGOMA): A multicentre, open-label, randomised, controlled, phase 2 trial. *Lancet Oncol.* **2019**, *20*, 110–119. https://doi.org/10.1016/S1470-2045(18)30675-2.
115. Gilbert, C.A.; Daou, M.-C.; Moser, R.P.; Ross, A.H. γ-Secretase Inhibitors Enhance Temozolomide Treatment of Human Gliomas by Inhibiting Neurosphere Repopulation and Xenograft Recurrence. *Cancer Res.* **2010**, *70*, 6870–6879. https://doi.org/10.1158/0008-5472.CAN-10-1378.
116. Chen, K.-T.; Lin, Y.-J.; Chai, W.-Y.; Lin, C.-J.; Chen, P.-Y.; Huang, C.-Y.; Kuo, J.S.; Liu, H.-L.; Wei, K.-C. Neuronavigation-guided focused ultrasound (NaviFUS) for transcranial blood-brain barrier opening in recurrent glioblastoma patients: Clinical trial protocol. *Ann. Transl. Med.* **2020**, *8*, 673. Available online: https://atm.amegroups.org/article/view/44421 (accessed on 1 January 2020).
117. Kirson, E.D.; Dbalý, V.; Tovaryš, F.; Vymazal, J.; Soustiel, J.F.; Itzhaki, A.; Mordechovich, D.; Steinberg-Shapira, S.; Gurvich, Z.; Schneiderman, R.; et al. Alternating electric fields arrest cell proliferation in animal tumor models and human brain tumors. *Proc. Natl. Acad. Sci. USA* **2007**, *104*, 10152–10157. https://doi.org/10.1073/pnas.0702916104.
118. Stupp, R.; Wong, E.T.; Kanner, A.A.; Steinberg, D.; Engelhard, H.; Heidecke, V.; Kirson, E.D.; Taillibert, S.; Liebermann, F.; Dbalý, V.; et al. NovoTTF-100A versus physician's choice chemotherapy in recurrent glioblastoma: A randomised phase III trial of a novel treatment modality. *Eur. J. Cancer* **2012**, *48*, 2192–2202. https://doi.org/10.1016/j.ejca.2012.04.011.
119. Stupp, R.; Taillibert, S.; Kanner, A.; Read, W.; Steinberg, D.M.; Lhermitte, B.; Toms, S.; Idbaih, A.; Ahluwalia, M.S.; Fink, K.; et al. Effect of Tumor-Treating Fields Plus Maintenance Temozolomide vs Maintenance Temozolomide Alone on Survival in Patients With Glioblastoma: A Randomized Clinical Trial. *JAMA* **2017**, *318*, 2306–2316. https://doi.org/10.1001/jama.2017.18718.
120. Miretti, M.; González Graglia, M.A.; Suárez, A.I.; Prucca, C.G. Photodynamic therapy for glioblastoma: A light at the end of the tunnel. *J. Photochem. Photobiol.* **2023**, *13*, 100161. https://doi.org/10.1016/j.jpap.2023.100161.
121. Bhanja, D.; Wilding, H.; Baroz, A.; Trifoi, M.; Shenoy, G.; Slagle-Webb, B.; Hayes, D.; Soudagar, Y.; Connor, J.; Mansouri, A. Photodynamic Therapy for Glioblastoma: Illuminating the Path toward Clinical Applicability. *Cancers* **2023**, *15*, 3427. https://doi.org/10.3390/cancers15133427.
122. Mitchell, M.J.; Billingsley, M.M.; Haley, R.M.; Wechsler, M.E.; Peppas, N.A.; Langer, R. Engineering precision nanoparticles for drug delivery. *Nat. Rev. Drug Discov.* **2021**, *20*, 101–124. https://doi.org/10.1038/s41573-020-0090-8.
123. Weller, M.; van den Bent, M.; Tonn, J.C.; Stupp, R.; Preusser, M.; Cohen-Jonathan-Moyal, E.; Henriksson, R.; Rhun, E.L.; Balana, C.; Chinot, O.; et al. European Association for Neuro-Oncology (EANO) guideline on the diagnosis and treatment of adult astrocytic and oligodendroglial gliomas. *Lancet Oncol.* **2017**, *18*, e315–e329. https://doi.org/10.1016/S1470-2045(17)30194-8.
124. Bloch, O.; Han, S.J.; Cha, S.; Sun, M.Z.; Aghi, M.K.; McDermott, M.W.; Berger, M.S.; Parsa, A.T. Impact of extent of resection for recurrent glioblastoma on overall survival: Clinical article. *J. Neurosurg. JNS* **2012**, *117*, 1032–1038. https://doi.org/10.3171/2012.9.JNS12504.
